# *GmCCD4* controls carotenoid content in soybeans

**DOI:** 10.1111/pbi.13506

**Published:** 2020-11-23

**Authors:** Jinshan Gao, Suxin Yang, Kuanqiang Tang, Guang Li, Xiang Gao, Bao Liu, Shaodong Wang, Xianzhong Feng

**Affiliations:** ^1^ Key Laboratory of Soybean Molecular Design Breeding Northeast Institute of Geography and Agroecology Chinese Academy of Sciences Changchun China; ^2^ Key Laboratory of Molecular Epigenetics of Ministry of Education (MOE) Northeast Normal University Changchun China; ^3^ Key Laboratory of Soybean Biology of Education Ministry Northeast Agricultural University Harbin China

**Keywords:** *GmCCD4* gene, genetic mapping, carotenoids, yellow flower, soybean

## Abstract

To better understand the mechanisms regulating plant carotenoid metabolism in staple crop, we report the map‐based cloning and functional characterization of the *Glycine max carotenoid cleavage dioxygenase 4* (*GmCCD4*) gene, which encodes a carotenoid cleavage dioxygenase enzyme involved in metabolizing carotenoids into volatile β‐ionone. Loss of GmCCD4 protein function in four *Glycine max increased carotenoid content (gmicc)* mutants resulted in yellow flowers due to excessive accumulation of carotenoids in flower petals. The carotenoid contents also increase three times in *gmicc1* seeds. A genome‐wide association study indicated that the *GmCCD4* locus was one major locus associated with carotenoid content in natural population. Further analysis indicated that the haplotype‐1 of *GmCCD4* gene was positively associated with higher carotenoid levels in soybean cultivars and accumulated more β‐carotene in engineered *E. coli* with ectopic expression of different GmCCD4 haplotypes. These observations uncovered that GmCCD4 was a negative regulator of carotenoid content in soybean, and its various haplotypes provide useful resources for future soybean breeding practice.

## Introduction

Carotenoids are widely distributed in plants, algae and bacteria, with more than 750 members in nature. Carotenoids are important for the human diet as they are the only precursors for vitamin A biosynthesis (Giuliano *et al*., 2008). In humans, carotenoids promote antioxidant activity and reduce age‐related macular degeneration of the eye (Davies, 2007; Fraser and Bramley, 2004; Krinsky and Johnson, 2005). Previous studies have shown that carotenoids dissolved in oil or aqueous dispersions are efficiently absorbed (>50%) (Blomstrand and Werner, 1967; Goodman *et al*., 1966), while carotenoids in uncooked vegetables, such as β‐carotene in carrots or lycopene in tomato juice, are poorly absorbed (<3%) (Chug‐Ahuja *et al*., 1993; Khachik *et al*., 1992; Stahl and Sies, 1992). Given the significance of carotenoids in the prevalence of vitamin A deficiency in developing countries, a better understanding of the mechanisms regulating plant carotenoid composition is essential, especially in edible seeds of staple crops (Chandler *et al*., 2013; Gonzalezjorge *et al*., 2013; Shewmaker *et al*., 1999).

Animals are unable to synthesize carotenoids *de novo* and must obtain these compounds via ingestion (Zhai *et al*., 2016). Plant carotenoid biosynthesis pathway has been elucidated from previous studies, Ɛ‐carotene desaturase (ZDS), carotenoid isomerases (ZISO and CRTISO) and cyclase (LCY) are four key regulatory enzymes of twelve enzymes in this pathway (Fantini *et al*., 2013; Nisar *et al*., 2015). ZISO, ZDS and CRTISO catalyse Ɛ‐carotene to all‐*trans*‐lycopene, the latter could be cyclized to α‐carotene and β‐carotene to originate two biosynthesis sub‐pathways of lutein and neoxanthin, respectively. α‐carotene is further hydroxylated by β‐OHases (encoded by *LUT5*) and Ɛ‐OHases (encoded by *LUT1*) to produce zeinoxanthin and lutein; β‐carotene is further hydroxylated by β‐Ohases to produce zeaxanthin (Cazzaniga *et al*., 2012; Kim *et al*., 2009). Zeaxanthin epoxidase (ZEP) hydroxylates the β‐rings of zeaxanthin to yield antheraxanthin and then violaxanthin; this reaction can be inverted by violaxanthin de‐epoxidase (VDE) (Hieber *et al*., 2000). Violaxanthin is converted to neoxanthin by neoxanthin synthase (NSY) in the final step of the core carotenoid biosynthetic pathway (Nisar *et al*., 2015).

Steady‐state carotenoid accumulation depends on the metabolic equilibrium between carotenoid biosynthesis/storage and carotenoid degradation in plants (Hannoufa and Hossain, 2012; Li and Yuan, 2013). Thus, the catalytic activity of carotenoid cleavage oxygenases (CCOs), which results in the enzymatic breakdown of C40 carotenoids into apocarotenoids, is critical for regulating carotenoid accumulation. The CCO exists in two forms, namely CCD (Carotenoid Cleavage Dioxygenase) and NCED (Nine‐Cis Epoxycarotenoid Dioxygenase) (Auldridge *et al*., 2006; Priya *et al*., 2019). In *Arabidopsis*, four CCDs (CCD1, CCD4, CCD7, CCD8) and five NCEDs (NCED2, NCED3, NCED5, NCED6, NCED9) were identified (Bouvier *et al*., 2005). CCD1 and CCD4 cleavage carotenoids to form volatile small apocarotenoids (Hou *et al*., 2016; Nacke *et al*., 2012; Pu *et al*., 2020; Yahyaa *et al*., 2015; Zhang *et al*., 2015; Zheng *et al*., 2019). CCD7/MAX3 and CCD8/MAX4 are involved in the synthesis of strigolactones (SLs) (Alder *et al*., 2012; Gomez‐Roldan *et al*., 2008; Wang *et al*., 2019; Wang *et al*., 2020). The five members of the NCED sub‐group are exclusively involved in cleavage of violaxanthin and neoxanthin to form ABA (Fantini *et al*., 2013).

Recent studies have increased our understanding of carotenoid metabolism, genetic regulation and genetic manipulation for high‐carotenoid cultivars in higher plants (Ashraf *et al*., 2015; Bai *et al*., 2016; Pu *et al*., 2020; Sui *et al*., 2016; Zeng *et al*., 2015; Zheng *et al*., 2019). Soybean (*Glycine max*) products are increasingly consumed by humans worldwide, as this protein‐ and oil‐dense food meets human nutritional needs and improves human living standards. While there are plenty of carotenoid metabolism pathways that remain to be learned in higher plants, the genetic underpinnings understood to date have been successfully translated to significantly elevate levels of carotenoids in soybean, transgenic seeds accumulated carotenoid 60–741 µg/gr seed by transforming the phytoene synthase (crtB) gene from *Pantoea ananatis* (Pierce *et al*., 2015), 845 µg/gr seed by overexpressing a seed‐specific bacterial phytoene synthase gene from *Pantoea ananatis* (Schmidt *et al*., 2014), over 800 µg/g seed by expressing the maize phytoene synthase and 500 µg/g seed by expressing the β‐carotene hydroxylase (CrtZ), β‐carotene ketolase (CrtW) genes from *Brevundimonas* sp., along with the maize phytoene synthase (Park *et al*., 2017), which the latter, includes the synthesis of the high value carotenoid astaxanthin in the seed. However, the function of soybean gene in carotenoid metabolism is still lacking. The only reported studies are limited to the correlation between the expression level of *GmCCD1*/*GmCCD4* and lutein content (Kanmaru *et al*., 2009), and the transcriptional response of carotenoid oxygenase genes to abiotic stresses (Wang *et al*., 2013). In this study, to assess the genetic control of carotenoid levels in soybean, a forward genetic strategy was employed to identify mutants with yellow flower and increased carotenoid content in a γ‐irradiated mutant population. We found that *GmCCD4* was a negative regulator of carotenoids accumulation in soybean, and the strong association between polymorphisms in the *GmCCD4* gene and carotenoid content among soybean cultivars. This work illustrated that *Glycine max carotenoid cleavage dioxygenase 4* (*GmCCD4*), encoding CCD4 in soybean, catalysed the cleavage of carotenoids to regulate the carotenoids turnover.

## Results

### Identification of soybean yellow flower mutants

**Figure 1:**
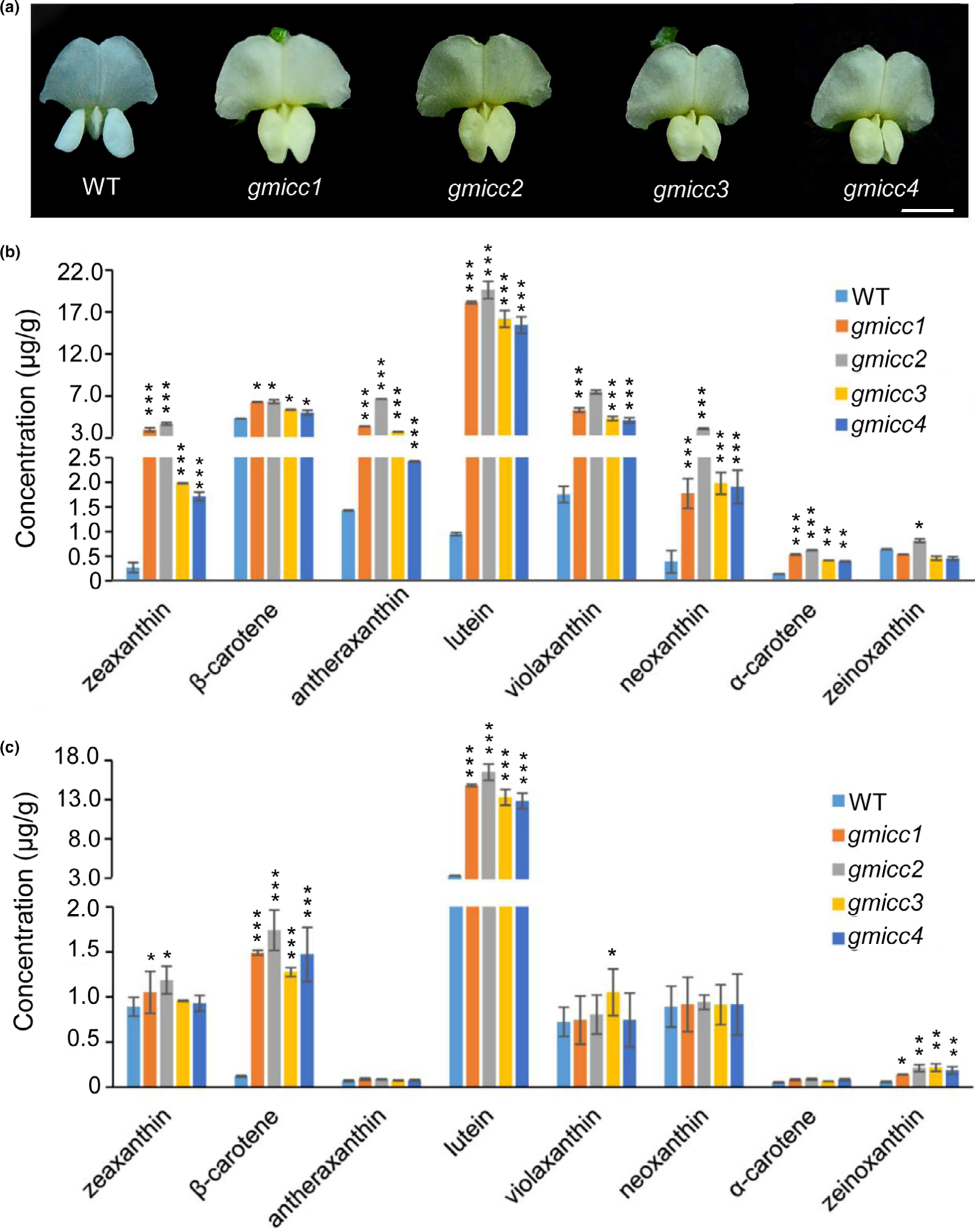
Phenotypes of the wild‐type (WT) and *gmicc* mutants. (a) Flowers of the WT and the *gmicc1*, *gmicc2*, *gmic3* and *gmicc4* mutants. Scale bar = 5 mm. (b) The carotenoid contents of the *gmicc1*, *gmicc2*, *gmicc3* and *gmicc4* flowers compared with the WT. (c) The carotenoid contents of the *gmicc1*, *gmicc2*, *gmicc3* and *gmicc4* seeds compared with the WT. Asterisks indicate statistically significant differences relative to the WT (*p < 0.05, **p < 0.01 and ***p < 0.001; Student's *t* test).

We screened 100,000 γ‐ray‐induced mutants, generated from Williams 82, for yellow flowers, and identified four mutants with flowers of different shades of yellow (Figure 1a). A slightly differences in seed coat or cotyledon colour were observed between the four mutants and WT (Figure S1). The mutants, in addition to yellow flowers, exhibited considerably increased carotenoid contents compared with the WT (Figure 1b,c). The four mutants were termed *Glycine max increased carotenoid content 1, 2, 3* and *4* (*gmicc1*, *2*, *3*, and *4*). The total carotenoid concentrations of the flowers and seeds in the *gmicc* mutants were over threefold and twofold higher than the WT, respectively (Table S1). Compared with WT, all kinds of carotenoids in flowers of *gmicc* mutants were significantly increased except zeinoxanthin, with 13‐fold increase in zeaxanthin, threefold in antheraxanthin, 19‐fold in lutein, threefold in violaxanthin, sixfold in neoxanthin and threefold in α‐carotene (Figure 1b). In comparison with WT, *gmicc* seeds had higher levels of β‐carotene, lutein and zeaxanthin in seeds, with 11‐fold increase in β‐carotene, threefold in lutein and twofold in zeinoxanthin (Figure 1c). The increased carotenoid contents in the *gmicc* seeds as compared to the WT were primarily due to increases in the β‐carotene and lutein concentrations (Figure 1b,c; Table S1). Genetic allelic tests were conducted by crossing each of these three mutants with the *gmicc1* mutant, and the all three types of intercrossed F1 hybrids exhibited the mutated yellow flower phenotype. This demonstrated that *gmicc1*, *2*, *3* and *4* were genetically allelic to each other (Table S2).

### Cloning and characterization of the candidate gene of *gmicc* mutants

In order to identify the mutation responsible for the observed phenotypic changes, Hedou 12, a Chinese soybean cultivar with purple flowers (Song *et al*., 2015), was crossed with the *gmicc1* mutant to obtain an F_2_ population of 976 individuals. Of these 976 plants, 706 exhibited the WT white flower phenotype and 270 exhibited the mutant yellow flower phenotype. The segregating 2.61:1 ratio between white and yellow flower phenotypes indicated that these phenotypic changes were controlled by a single recessive locus (fitting the 3:1 ratio of one gene mutation segregation, χ^2^ test, *p* = 0.18).

**Figure 2:**
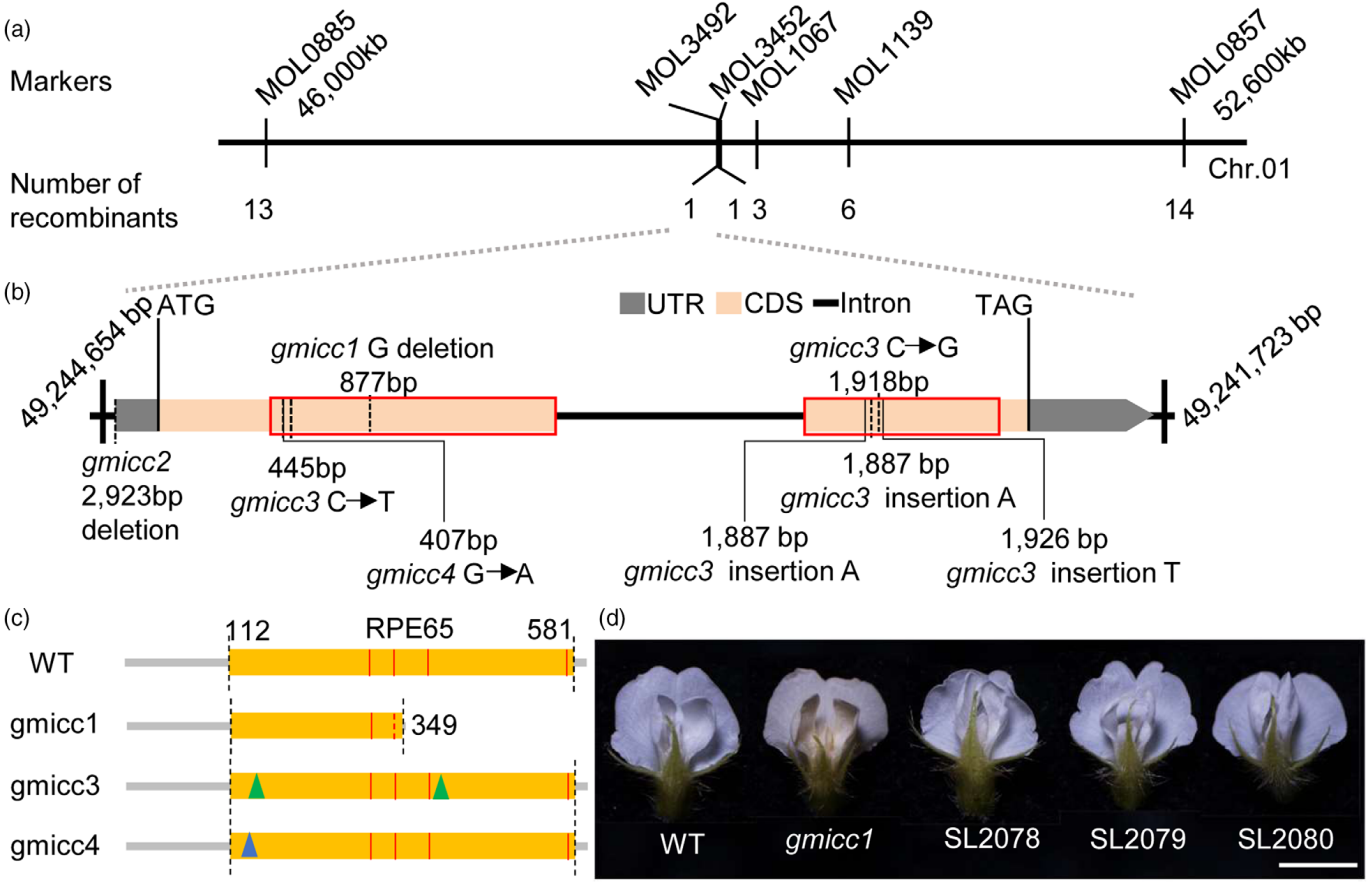
Map‐based cloning of the candidate locus. (a) Physical locations of the markers defining the candidate region. The chromosomal positions of MOL3492 and MOL3452 were 49,241.7 kb and 49,244.6 kb, respectively (*Glycine max Wm82.a2.v1*). (b) The structure of the *Glyma.01G154900.1* gene. Red boxes indicate the RPE65 domain in the ORF. A single nucleic acid deletion was detected in *Glyma.01G154900.1* of the *gmicc1* mutant. A large deletion abolishing *Glyma.01G154900.1* in the *gmicc2* mutant was identified. Two SNPs and three insertions in the *gmicc3* mutant were detected. A SNP in the *gmicc4* mutant was identified. (c) Structure of the wild‐type Glyma.01G154900.1 (WT) and mutant proteins in the *gmicc* mutants. Red lines indicate the locations of the four predicted catalytic sites in the RPE65 domain at amino acids 286, 335, 399 and 576. The gmicc1 protein is truncated due to a stop codon resulting from a frameshift mutation in the *gmicc1* mutant. The gmicc3 protein has 15 amino acids substitutes (green arrows) in the *gmicc3* mutant. The gmicc4 protein has one amino acid substitute (blue arrow) in the *gmicc4* mutant. (d) Complementation of the *gmicc1* mutant. Phenotypes of the WT, *gmicc1* mutant and transgenic complementation (SL2078, SL2079, SL2080; derived from the *gmicc1* mutant) plants. Scale bar = 5 mm.

The candidate locus was mapped using 270 F_2_ individual mutants with the mutant yellow flower phenotype. Using 165 previously developed INDEL markers (Song *et al*., 2015), the candidate locus was mapped to a genomic region between MOL0885 and MOL0857 on Chromosome 1; this region is 6.6 Mb in the soybean reference genome sequence (*G. max Wm82.a2.v1*) (Schmutz *et al*., 2010) (Figure 2a). The candidate locus was further located in a 3‐kb region between MOL3492 and MOL3452, with 27 recombinants (Figure 2a; Table S3). This region harbours one gene, *Glyma.01G154900.1*, based on the WT reference genome (*G. max Wm82.a2.v1*). This gene and its flanking sequences were sequenced within the defined *Glyma.01G154900.1* region of the WT and *gmicc1* mutant, and a single nucleotide deletion in the coding region of *Glyma.01G154900.1* (the 877th G in the first exon) was identified (Figure 2b). This deletion caused a frameshift leading to a premature stop codon and truncated protein (Figure 2c). The Glyma.01G154900.1 protein contains a RPE65 domain, which was affected by the mutation in the mutant gmicc1 protein and caused the loss of two of the four catalytic sites (Figure 2c). The mutations did not cause significant alterations in the expression levels of the *Glyma.01G154900.1* gene in the flowers of the *gmicc* mutants except *gmicc2* (Figure S2a) suggesting that disruption in the protein function caused the *gmicc* mutant phenotype.

These results suggested that *Glyma.01G154900.1* was the candidate gene. In order to verify this supposition, the genomic regions of the *Glyma.01G154900.1* gene in the *gmicc1*, *2*,*3* and *4* mutants were amplified (Figure S2b). In the *gmicc2* mutant, there was a large deletion abolishing the entire *Glyma.01G154900.1* gene. In the *gmicc3* mutant, there were two single nucleotide mutations (from C to T at position + 445 bp and from C to G at position + 1,918 bp) and three single nucleotide insertions (the A at position + 1,887 bp, A at position + 1,897 bp and T at position + 1,926 bp), which leading to 15 amino acids substitutes (Figure 2b,c). In the *gmicc4* mutant, there was a single nucleotide mutation (from G to A at position + 407 bp), which leading to one amino acid substitute in the Retinal Pigment Epithelium 65 kDa protein (RPE65) domain (Figure 2b,c) (Chander *et al*., 2012; Kiser *et al*., 2012; Kloer, 2005; Priya *et al*., 2016). All these mutations caused either complete loss of the entire protein or functional loss of the Glyma.01G154900.1 protein (Figure 2c). In combination, the sequencing results and the allelism tests revealed that independent mutations in *Glyma.01G154900.1* always resulted in yellow flowers and increased carotenoid content.

In order to confirm these results, a 7 kb genomic fragment, including 3 kb upstream and 1 kb downstream sequences of the *Glyma.01G154900.1* gene, was introduced into the *gmicc1* mutant using *Agrobacterium tumefaciens*‐mediated transformation (Yamada *et al*., 2010). Three independent complementation transgenic lines in the *gmicc1* background exhibited the white flower colour and reduced carotenoid contents in both seeds and flowers same as WT (Figure 2d, Figure S3a, b and Table S1). These transgenic plants were verified by detection of inserted genes (Figure S3c,d), bar protein immunoassay (Figure S3d) and exogenous transcripts (Figure S3e). The transcripts of both exogenous transformed wild‐type and mutated *gmicc1* were found in these transgenic plants in the *gmicc1* background (Figure S3e), and there was no significant difference in the expression levels of the *Glyma.01G154900.1* among WT, *gimicc1* and transgenic plants (Figure S3f). This result also demonstrated that *Glyma.01G154900.1* was indeed the candidate gene.

### The mutated candidate gene encoded GmCCD4

**Figure 3:**
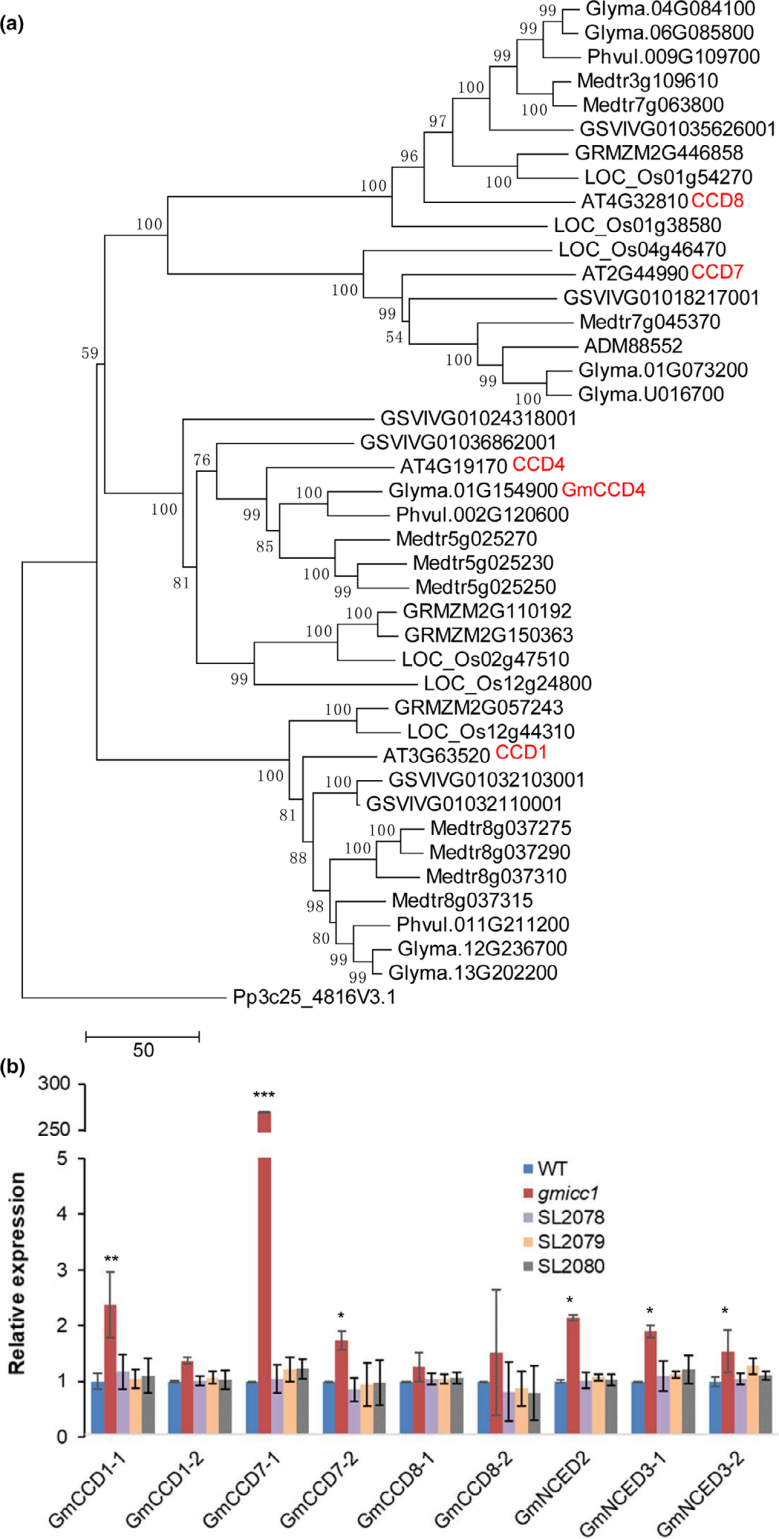
Phylogenetic analysis of CCD proteins and *GmCCD* gene expression patterns. (a) NJ phylogeny of CCD homologs from *Arabidopsis thaliana*, grape (*Vitis vinifera*), soybean (*Glycine max*), green bean (*Phaseolus vulgaris*), medicago (*Medicago truncatula*), crowtoe (*Lotus japonicus*), rice (*Oryza sativa*), corn (*Zea mays*) and moss (*Physcomitrella patens*). Numbers at nodes are bootstrap support values (1000 replicates); scale bar represents the number of amino acid substitutions per site. (b) Relative expression of *GmCCDs* in the opened flowers of the WT, *gmicc1* mutant and transgenic complementation plants (SL2078, SL2079, SL2080). Details of the 10 *GmCCD* genes detected in Figure 3b are given in Table S1. Expression levels are presented as the mean ± SD (standard deviation) of three biological replicates. Asterisks indicate statistically significant differences relative to the WT (*p < 0.05, **p < 0.01 and ***p < 0.001; Student's *t* test).

Previous bioinformatic study assigned the *Glyma.01G154900.1* gene as *GmCCD4* in soybean (Kanmaru *et al*., 2009), we followed this pre‐existing name as the target gene of our mutants in this study. In order to determine the phylogenetic relationships and functional conservation of this protein across different species, 42 homologs of the *AT4G19170* (*CCD4*) gene from nine species were aligned and used to construct a neighbour‐joining (NJ) phylogenetic tree (Table S4). The phylogenetic analysis of the GmCCD4 and GmCCD4‐like proteins revealed that *GmCCD4* was most likely a single copy gene with high identity to the *Arabidopsis CCD4* gene (Figure 3a). A synteny analysis of the 87,000 bp region surrounding *AtCCD4* (beginning at 10,450,000 bp and ending at 10,537,000 bp on *A. thaliana* chromosome 4) was performed against the *G. max* genome using the webtool MCScanX (Wang *et al*., 2012). The synteny plot showed that the gene arrangement of the *AtCCD4* flanking sequence was highly syntenic to the gene arrangements of *G. max* chromosomes 1 and 11. However, the *GmCCD4* homologue was lost from the homologous region of chromosome 11 (Figure S4a). Putative copies of *GmCCD4* were searched for in wild and cultivar soybean genomes and proteomes (Kim *et al*., 2010; Li *et al*., 2014; Zhou *et al*., 2015); these searches revealed that *GmCCD4* was a single copy gene among these sequenced soybeans.

The gene expression levels of *GmCCD4* were quantified in both vegetative and reproductive organs of the WT plants, including the roots, stems, leaves, stem apical meristems (SAMs), inflorescences, unopened flowers, opened flowers, pods and seeds using real‐time quantitative PCR (qPCR). *GmCCD4* was most highly expressed in opened flowers, followed by pods and unopened flowers, while *GmCCD4* gene was expressed the lowest in roots (Figure S4b). Besides the seven carotenoid cleavage dioxygenases, three 9‐cis epoxycarotenoid dioxygenases were identified in the soybean (Figure S5). The expression levels of the carotenoid cleavage dioxygenases and the 9‐cis epoxycarotenoid dioxygenases were detected in the opened flowers of the WT, *gmicc1* mutant and transgenic complementation lines. Of the nine *GmCCD‐like* genes, *GmCCD1‐1*, *GmCCD7‐1*, *GmCCD7‐2*, *GmNCED2*, *GmNCED3‐1* and *GmNCED3‐2* were the most highly expressed in opened flowers of the *gmicc1* mutant compared with the WT and complementation lines (Figure 3b). The significant increase of *GmCCD7‐1* expression might due to the substrate similarity between GmCCD4 and GmCCD7‐1. This suggested that lack of *GmCCD4* function induced the expression of other *GmCCD*‐like genes in the *gmicc1* mutant.

### GmCCD4 catalysed the degradation of β‐ and/or α‐carotenes

**Figure 4:**
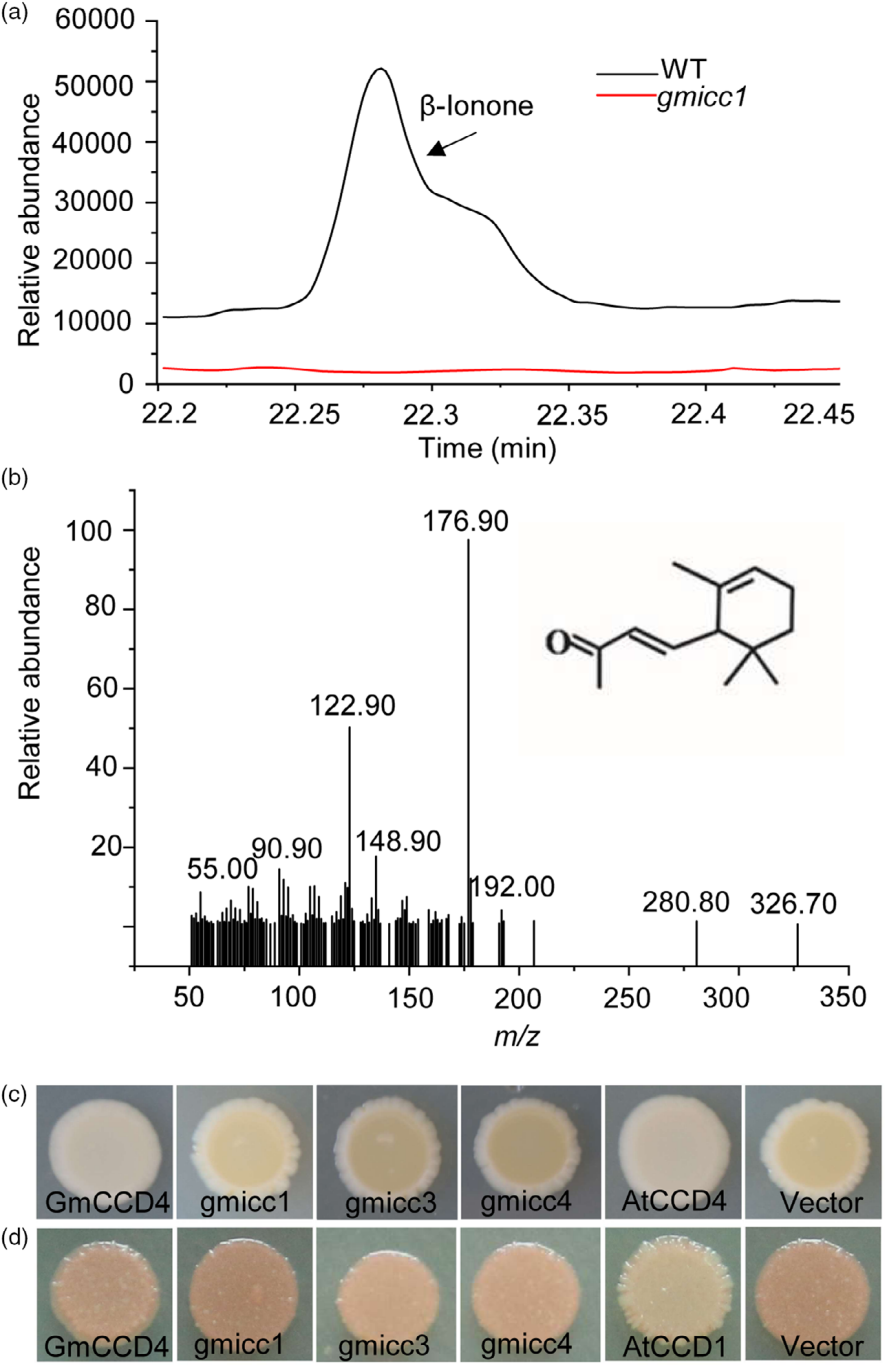
Enzymatic characteristics of GmCCD4. (a) Headspace solid‐phase microextraction GC‐MS analysis of volatiles released from the flower petals of WT and *gmicc1* plants. (b) Mass spectrum of β‐ionone. (c) Bacterial colonies harbouring the carotenoid biosynthetic genes encoding β‐carotene (*pACCAR16ΔcrtX*) and co‐expression of *GmCCD4*, *gmicc1*, *gmicc3*, *gmicc4*, *AtCCD4* and the pET32a empty vector. (d) Bacterial colonies harbouring the carotenoid biosynthetic genes encoding lycopene (*pACCRT‐EIB*) and co‐expression of *GmCCD4*, *gmicc1*, *gmicc3*, *gmicc4*, *AtCCD1* and the ET32a empty vector.

Most plant CCD4s catalyse the oxidative cleavage of carotenoids into volatile apocarotenoids at the double‐bond position 9, 10 (9’, 10’) (Huang *et al*., 2009; Rubio *et al*., 2008; Zhang *et al*., 2015). In order to determine the cleavage reaction of carotenoids catalysed by GmCCD4, volatile apocarotenoids were analysed in the petals of the WT, *gmicc1* mutant and complementation lines. A major peak, with a retention time of 14.42 min, was detected in the volatile compounds released from the white petals of the WT and T_2_ complementation lines (Figure 4a; Figure S6a‐c); the mass spectrum of this peak was consistent with that of β‐ionone (Figure 4b). In contrast, the yellow petals of the *gmicc1* mutant did not release β‐ionone (Figure 4a).

In order to investigate the enzymatic characteristics of GmCCD4 and mutation proteins, *GmCCD4*, *gmicc1*, *gmicc3* and *gmicc4* genes were introduced into *Escherichia coli* strains previously engineered to accumulate β‐carotene. The yellow colour was retained in *E. coli* cells transformed with the pET32a‐gmicc1, pET32a‐gmicc3 and pET32a‐gmicc4, while the yellow colour was lost in *E. coli* cells transformed with pET32a‐GmCCD4 (Figure 4c). β‐carotene accumulated in *E. coli* cells carrying the mutated GmCCD4 proteins, indicating that gmicc proteins did not cleave this carotenoid. In contrast, lycopene accumulated in *E. coli* carrying either GmCCD4 or gmicc (Figure 4d), indicating that neither of these proteins cleaved lycopene. These results indicated that *GmCCD4* encodes a functional CCD enzyme and the yellow flower phenotype of the *gmicc* mutants was caused by the inhibition of CCD enzymatic activity.

GC‐MS analysis of the headspace of *E. coli* cells harbouring *GmCCD4* and *gmicc1* indicated that these cells generated β‐methylionone. In addition, β‐methylionone was present only in cells harbouring *GmCCD4*; β‐methylionone was absent in cells harbouring *gmicc1* and the pET32a plasmid (Figure S6d–f). Based on the chemical structures of β‐ and/or α‐carotenes, the β‐ionone released in the WT and complementation lines were the product of β‐ and/or α‐carotenes cleaved preferentially at the double‐bond position 9, 10 by GmCCD4 (Figure S6g). We speculated that the generation of β‐methylionone in *E. coli* cells might have been the result of the microbial transformation of β‐ionone in bacteria.

### Genome‐wide association study of carotenoids and variations of *GmCCD4*

**Figure 5:**
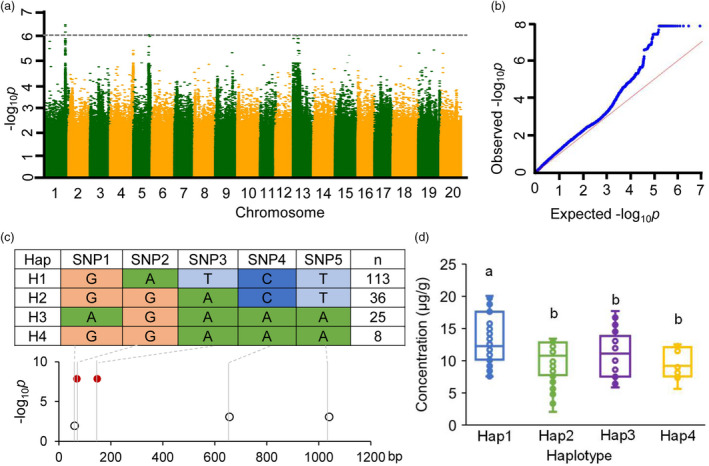
GWAS of β‐carotenoid content using EMMAX and haplotype analyses at the *GmCCD4* locus. (a) Manhattan plots for β‐carotenoid content. Negative log_10_ *p*‐values from a genome‐wide scan are plotted against SNP positions of 20 chromosomes. The horizontal dash line indicates the threshold of significance (7.66×10^‐^^7^). (b) Quantile–quantile plot for β‐carotenoid content. (c) Haplotype analysis of *GmCCD4* in the sequence of 182 cultivated soybeans (unpublished data). SNP1, SNP2, SNP3, SNP4 and SNP5 are non‐synonymous mutations located at 49,244,470, 49,244,461, 49,244,383, 49,243,874 and 49,243,481 positions of Chromosome 1, respectively. Sample number (*n*) is the number of germplasm lines under each haplotype. Black circles represent mutations not associated with the phenotypic transition. Solid red circles represent causative mutations associated with the phenotypic transition. (d) β‐carotene content of different *GmCCD4* haplotypes. Box edges indicate the interquartile range; whiskers indicate 1.5 × the interquartile range, and centre lines indicate the median. Significant differences among groups were identified using one‐way ANOVAs, followed by Tukey’s multiple comparisons post hoc tests (*p* < 0.05). Different letters indicate distinct groups.

A genome‐wide association study (GWAS) of carotenoid contents with four million markers generated through genome‐resequencing among 182 cultivars was conducted (Table S5). The distribution of β‐carotenoid contents among these cultivars looked approximately normal and seemed appropriate for GWAS (Figure S7). As shown in the Manhattan plots and quantile–quantile for β‐carotenoid content, we found 3 notable positive association (*p *≤ 7.66×10^‐^^7^ in the standard mixed linear model) loci, including the *GmCCD4* locus (Figure 5a,b). Further analysis showed that the *GmCCD4* locus explained about 16.84% of the variance in β‐carotenoid content across the entire population of 182 cultivars. Of the SNPs identified in the *GmCCD4* locus, 11, including five non‐synonymous, were located in the exon region, one was located in the 5’UTR, and 20 were located in the intron region (Table S6 and S7). These results further demonstrated that *GmCCD4* was a possible candidate gene of genetic variation in carotenoid content across the examined soybean germplasms.

To further investigate whether *GmCCD4* polymorphism was related to the carotenoid content, we identified four haplotypes among the 182 soybean germplasms, based on the five non‐synonymous SNPs in the *GmCCD4* locus (Figure 5c). These five SNPs were located in the first exon of *GmCCD4* gene, SNP1 and SNP3 caused significant hydrophobicity changes among haplotypes (Table S7), which could influence various noncovalent bonds, such as the interaction between enzyme and substrate, antibody, and antigen (Bloemendal *et al*., 1989). Even though SNP4 and SNP5 were in the PRR65 domain, but the hydrophobicity changes were smaller than SNP1 and SNP3 (Table S7). The hydrophobicity change of SNP2 was similar as SNP4 and SNP5; however, it was co‐segregated with SNP3. Among these five non‐synonymous SNPs, SNP2 and SNP3 were positively associated with higher carotenoid content in seeds (Figure 5c). According to SNP2 and SNP3 polymorphisms, the four haplotypes were divided into two subgroups, H1 and H2/3/4. Among H2/3/4, the characteristic polymorphism variation of H2 was SNP4 and SNP5, and H3 was SNP1, respectively (Figure 5c). Interestingly, large proportions of the cultivars carried haplotype 1 and had higher levels of β‐carotenoids (Figure 5d; Figure S8).

### Analysis of genotype and phenotype relationship of *GmCCD4*

**Figure 6:**
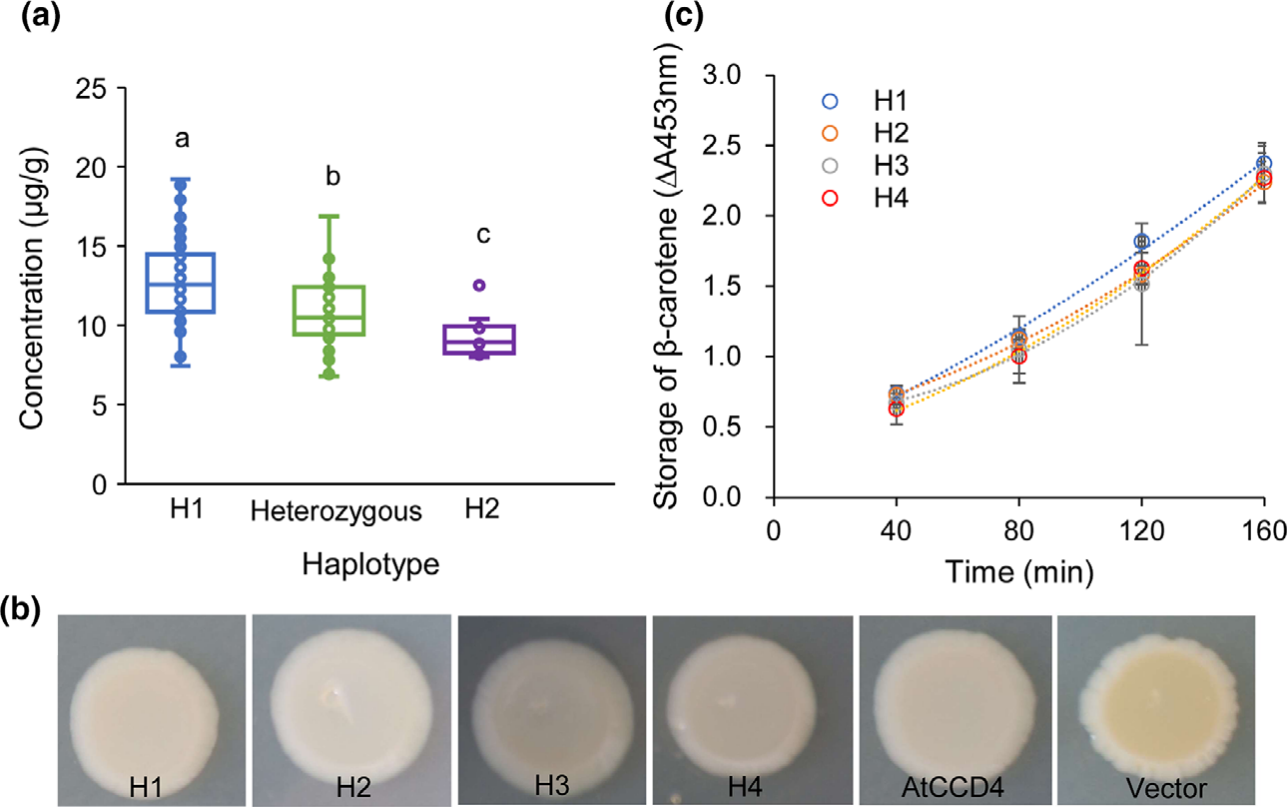
β‐carotene content of the F_2_ population and enzymatic characteristics of different haplotype of GmCCD4. (a) β‐carotene contents in different *GmCCD4* haplotypes. Box edges depict the interquartile range, whiskers indicate 1.5 × the interquartile range, and centre lines represent the median. Significance was calculated by one‐way ANOVA followed by Tukey’s multiple comparisons post hoc test (*p* < 0.05). Different letters indicate distinct groups. (b) Bacterial colonies harbouring the carotenoid biosynthetic genes encoding β‐carotene (*pACCAR16ΔcrtX*) and co‐expression of four haplotype *GmCCD4* (H1, H2, H3 and H4), *AtCCD4*, and the pET32a empty vector. (c) Enzymatic characteristics of different haplotypes of GmCCD4. Absorbance at 453 nm was measured every 40 min.

Pedigree linkage analysis were also performed in an F_2_ population that was derived from a cross between low‐carotenoid line Heinong 46 (haplotype 2) and high‐carotenoid line Hefeng 39 (haplotype 1). A total 119 F_2_ individuals were collected for genotyping and phenotyping. The F_2_ plants were genotyped as three groups according to the *GmCCD4* genotype: haplotype 1, haplotype 2, and heterozygous. The β‐carotene contents of plants in the different groups were significantly different. That is, the F_2_ individuals carrying haplotype 1 had the highest levels of β‐carotene, while the F_2_ individuals carrying haplotype 2 had the lowest levels of β‐carotene; the heterozygous F_2_ individuals had intermediate levels of β‐carotene (Figure 6a). These results further indicated that haplotype 1 of GmCCD4 was positively associated with higher carotenoid content.

To investigate the enzymatic characteristics of the GmCCD4 proteins in cultivars with different haplotypes, *GmCCD4* genes with different haplotype were cloned from cultivars Heihe 43 (H1), Heinong 67 (H2), Hefeng 7 (H3) and Hudou 9765 (H4), and introduced into *E. coli* strains previously engineered to accumulate β‐carotene. The yellow colour was retained in *E. coli* cells transformed with the pET32a vector while the yellow colour was lost in *E. coli* cells transformed with four haplotypes of GmCCD4 (Figure 6b). Absorbance was measured every 40 min at 453 nm to monitor changes in β‐carotene levels. The *E. coli* cells transformed with haplotype‐1 of *GmCCD4* accumulated more β‐carotene than cells transformed with any of the other three *GmCCD4* haplotypes (Figure 6c). This indicated that haplotype‐1 of GmCCD4 exhibited the lowest enzymatic activity in degrading β‐carotene. However, the enzyme activities of four different haplotypes of GmCCD4 showed no significant difference *in vitro*. These observations indicated that four kinds of *GmCCD4* encode functional CCD4 enzyme in cleaving β‐carotene with no significant changes in enzyme activity *in vitro*.

### *GmCCD4* affected the dynamic equilibrium between carotenes and xanthophylls

**Figure 7:**
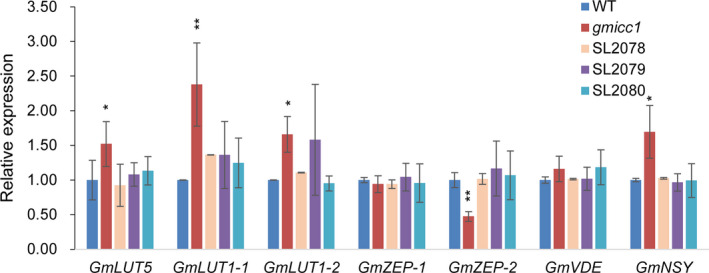
Expression patterns of genes associated with carotenoid biosynthesis genes in the WT, *gmicc1* mutant and transgenic complementation plants (SL2078, SL2079, SL2080). Expression levels are presented as the mean ± SD of three biological replicates. Asterisks indicate statistically significant differences relative to the WT (*p < 0.05 and **p < 0.01; Student's t test).

The expression of *GmLUT5* and *GmLUT1* increased 50% and 130% in the *gmicc1* mutant compared with the WT and transgenic complementation lines (Figure 7). The expression of *GmZEP‐2* decreased 122% in the *gmicc1* mutant compared with the WT and transgenic complementation lines; the expression level of *GmZEP‐1* and *GmVDE* exhibited no significant changes between the *gmicc1* mutant and WT (Figure 7). The expression of *GmNSY* increased 40% in the *gmicc1* mutant compared with the WT and transgenic complementation lines (Figure 7). It suggested that accumulation of antheraxanthin and violaxanthin in mutant might due to the expression changes of *GmZEP‐2* and *GmNSY* or the mutation of *GmCCD4*. Nevertheless, the metabolites of carotenoids were preferentially influenced by the mutation of *GmCCD4*.

**Figure 8:**
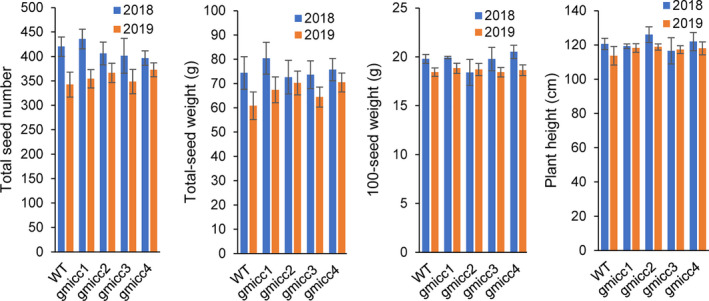
Yield components between the WT ang the *gmicc* mutants. No apparent changes in the yield or yield components between the WT and the *gmicc1*, *2*, *3* and *4* mutants. Values represent the mean ± SD of five different plants.

Furthermore, there were no significant differences in yield indices (i.e. seed number, plant height, seed weight and plant height) between the WT and *gmicc* mutants (Figure 8). These observations indicated that losing the function of GmCCD4 had limited influences on yield and other metabolite pathways, therefore, the *GmCCD4* gene could be a candidate bioengineering gene used for the propagation and proliferation of more nutritional soybeans.

## Discussion

Carotenoids endow flowers and fruits with distinct colours (e.g. yellow, orange and red colours) (Grotewold, 2006), which then attract animals for pollination or seed dispersal (Kevan and Baker, 1983). Carotenoids are also photo protectants, antioxidants and accessory pigments in photosynthesis (Grotewold, 2006; Walter and Strack, 2011). Compared with other members of the CCD family, disruption of CCD4 often causes colour variations in flowers, fruits and tubers. The functional *CCD4* gene is likely to be the major determinant in flower colour related to carotenoid accumulation (Brandi *et al*., 2011; Campbell *et al*., 2010; Ohmiya *et al*., 2009; Ohmiya *et al*., 2012). In *Chrysanthemum*, *CmCCD4a* is specifically transcribed in flowers; loss of function of CmCCD4 leads to the change of flower colour from white to yellow (Ohmiya *et al*., 2006). In *Brassica*, four variations, including two INDELs and two insertions of transposable elements in the *Bnac3.CCD4*, disrupt the function of BnaC3.CCD4 and change the petal colour from white to yellow (Zhang *et al*., 2015). In *Lonicera japonica*, *LjCCD4* plays important roles in dynamic flower coloration by cleaving carotenoids to apocarotenoids, and the expression pattern of *LjCCD4* is negatively correlated with the carotenoid content (Pu *et al*., 2020). CCD4s also contribute to the divergence of yellow and white colour in potato tubers and peach fruit flesh (Brandi *et al*., 2011; Campbell *et al*., 2010; Fantini *et al*., 2013). Consistent with previous studies, loss of GmCCD4 protein function in four *gmicc* mutants resulted in yellow flower colour in our study.

Until now, we firstly report yellow flower mutants in soybean, which are not existed in nature. Our four yellow flower mutants were selected from our laboratory 100,000 soybeans mutant population during the past ten years, we speculate that the huge amount of mutant screening is one of important reasons to find this novel phenotype. Interestingly, we also notified that some of the cultivars with much higher β‐carotene than *gmicc* mutants in seeds, but their flowers remain white colour. This implies that different regulation networks of carotenoid turnover might be employed in flower and seed. The conflict of flower colour and seed carotenoids accumulation was also observed in Arabidopsis, the petals of the knockout mutant *ccd4‐1* still kept white colour (personal correspondence from Professor Dean DellaPenna), even though the carotenoid content in seeds of the *ccd4‐1* mutant increased relative to the wild type (Gonzalezjorge *et al*., 2013). Besides *GmCCD4* locus, two other loci were also detected associating with seed carotenoid content in GWAS analysis experiment, which indicated that at least three regulators are involved in seed carotenoid content regulation. This suggests that various factors are involved in turnover of carotenoid in different organs, and the yellow flower phenotype might also appear in specific genetic background.

In C. *sativus*, the transcriptome analysis indicates that various TFs in carotenoid‐accumulating tissues possibly participate in the regulation of *CsCCD* expression in a TF‐dependent manner (Bouvier *et al*., 2003; Sui *et al*., 2016). In citrus, a 5’ *cis*‐regulatory change at *CCD4b* is a major genetic determinant of natural variation in C30 apocarotenoids responsible for red coloration of citrus peel (Zheng *et al*., 2019). Based on five non‐synonymous SNPs, four haplotypes of *GmCCD4* were identified among cultivars, which is tightly related to the carotenoid content. However, the enzyme activities of four different haplotypes of *GmCCD4* showed no significant difference *in vitro*. This suggests that these SNP polymorphisms could not directly regulate the enzyme activity of *GmCCD4* to control the accumulation of carotenoids, which might be likely through response to some *cis*‐ and *trans*‐factors as above. It is worthy to investigate the regulation network of carotenoid turnover mechanism in soybean in the future, which could bring more target genes for soybean improvement in the future.

## Experimental procedures

### Plant materials, DNA extraction and genetic mapping

Two soybean cultivars Hedou 12 and Williams 82, the WT, were obtained from the Chinese Academy of Agricultural Sciences (Beijing, China). Four *gmicc* mutants were identified by screening for yellow flowers among 100,000 WT γ‐irradiated mutants (Cheng *et al*., 2016). The plant heights were measured at R8 stage; the seed number per plant, total‐seed weight and weight of hundred seeds were measured after harvesting. Genomic DNA was extracted from both cultivars and from *gmicc* mutants using a DNeasy Plant Mini Kit (Qiagen, CA). Anchor markers used for primary mapping were retrieved from Song *et al*. (2015). Fine mapping markers of the *GmCCD4* region were developed using INDELs between Hedou 12 and WT following previously described methods (Song *et al*., 2015). All primers used are provided in Table S3.

### Plasmid construction and transformation

The *GmCCD4* gene (including 3 kb upstream and 1 kb downstream sequences) was amplified using the primers OL6826F and OL6826R from WT and inserted into the pCAMBIA3301 (Cambia) vector between restriction endonuclease *Sac*I and *Bam*HI sites to generate the *ProGmCCD4: GmCCD4* plasmid. The *ProGmCCD4: GmCCD4* plasmid was introduced into *Agrobacterium tumefaciens* strain EHA105 and then transformed to *gmicc1* mutants following the *Agrobacterium*‐mediated transformation protocol previously described by Yamada *et al*. (2010). PAT/bar quick test was conducted according the manufacturer (PAT/bar EPSPS LFD Strips, Youlong Biotech, China).

### Phylogenetic analysis

CCD protein sequences similar to the GmCCD4 enzyme were identified in various plant species using Phytozome V12 (Schmutz *et al*., 2010). In total, 42 peptide sequences were selected and aligned using CLUSTALW (Thompson *et al*., 1994). All positions with <5% alignment gaps, missing data and ambiguous bases were tolerated at any position. The final alignment had 309 positions. Then, an NJ phylogenetic tree was constructed based on this alignment using MEGA7 (Kumar *et al*., 2016; Saitou and Nei, 1987). The bootstrap consensus tree, inferred from 1,000 replicates, represented the evolutionary history of the taxa analysed (Felsenstein, 1985). Branches corresponding to partitions reproduced in ≤ 50% of all bootstrap replicates were collapsed. Evolutionary distances were computed using the Poisson correction method (Zuckerkandl and Pauling, 1965).

### RNA isolation and real‐time quantitative PCR analysis

Total RNA was isolated from soybean SAMs, leaves, stems, roots, inflorescences, unopened flowers, opened flowers, pods and seeds using TRNzol (TIANGEN, Beijing, China) following the manufacturer's instructions. cDNA was synthesized using a FastQuant RT Kit (with gDNase) (TIANGEN, Beijing, China), following the manufacturer's instructions. Relative gene expression was quantified with RT‐qPCR using a FastStart Universal SYBR Green Master (ROX) (Roche, Mannheim, Germany) in a Stratagene Mx3005P sequence Detection System (Applied Biosystems, Waldbrann, Germany) following the manufacturer's instructions. Three biological replicates were analysed to quantify the levels of gene expression, and three technical replicates were performed. Relative gene expression was calculated using the ATP‐binding cassette transporter gene (*Cons4*, *Glyma.12G020500*) as a control (Liu *et al*., 2016; Ping *et al*., 2014).

### Co‐expression of *GmCCD4* and carotenoid biosynthetic enzymes in *E. coli*

The full‐length cDNAs of *GmCCD4*, *gmicc1*, *gmicc3* and *gmicc4* were amplified from the WT, *gmicc1*, *gmicc3* and *gmicc4* mutants, respectively, using the primers OL6116F and OL6116R (containing the restriction enzyme sites *Eco*RI and *Hind*III, respectively). Amplified cDNAs were digested and cloned into the pET32a expression vector. The full‐length cDNAs of *CCD4* and *CCD1* were amplified from the flowers of *Arabidopsis* Col‐0. *CCD4* was amplified with the primers, OL6117F and OL6117R, which contained the restriction enzyme sites *Eco*RI and *Sal*I, respectively. *CCD1* was amplified with the primers, OL6118F and OL6118R, which contained the restriction enzyme sites *Eco*RI and *Bam*HI, respectively. Amplified cDNAs were digested and cloned into the pET32a expression vector for expression in *E. coli* strains.

GmCCD4/gmicc1/gmicc3/gmicc4/AtCCD4/AtCCD1 and the carotenoid biosynthetic enzymes, β‐carotene‐pACCAR16ΔcrtX and lycopene‐pACCRT‐EIB, were co‐expressed following previously described methods (Huang *et al*., 2009; Tian *et al*., 2014) with slight modifications. Briefly, the plasmids of the pET32a vector (pET32a‐*AtCCD4*, pET32a‐*AtCCD1*, pET32a‐*GmCCD4*, pET32a‐*gmicc1*, and pET32a‐*gmicc3* and pET32a‐*gmicc4*) were introduced into *E. coli* strain, BL21 (DE3), which had been previously transformed with the carotenoid biosynthetic genes encoding β‐carotene (*pACCAR16ΔcrtX*) or lycopene (*pACCRT‐EIB*) (Misawa *et al*., 1995). The *E. coli* strain was inoculated in 100 mL of Luria‐Bertani (LB) medium and cultured at 37 °C until the optical density at a wavelength of 600 nm (OD_600_) reached 0.4–0.6. Then, 1 mM isopropyl‐β‐D‐thiogalactopyranoside was added to LB solid medium and recombinant proteins were induced at 28°C for 4 h. *GmCCD4* genes with different haplotype were cloned from cultivars Heihe 43 (H1), Heinong 67 (H2), Hefeng 7 (H3) and Hudou 9765 (H4), introduced into *Escherichia coli* strains and expressed.

### Extraction and quantification of carotenoids

In order to measure carotenoid concentration, seeds and flower petals were weighed, freeze‐dried for 17.5 h and ground with a Mixer Mill MM 400 (Retsch, Haan, Germany). Carotenoids were extracted following previously described methods (Yang *et al*., 2015). Then, the compounds were separated in 10 µL aliquots extract using an Exion UPLC coupled to a QTRAP 6500 PLUS (Sciex) equipped with a Luna silicagel column (3 μm; 150 mm × 2.0 mm) (Phenomenex) at 40°C. The mobile phases were acetonitrile (ACN):methanol:dichloromethane (80:15:5 [v:v:v]; A) and ACN:methanol:dichloromethane (30:20:50 [v:v:v]; B), with a flow rate of 0.8 mL/min at the following gradient: 0–18 min, 5% buffer B increased to 70%; 19–20 min, buffer B reduced to 5%; and 20–22 min, buffer B held at 5%. Compounds in the carotenoids were identified based on characteristic absorption spectra, typical retention times and standards published by CaroNature Co. (Bern, Switzerland). Carotenoids were quantified with standard calibration curves, and cholesterol‐d6 was used as an internal recovery control. The β‐carotenoid content in cultivars was calculated using the absorbance value measured in a Nanophotometer (IMPLEM, Germany) at 453 nm.

### Analysis of volatile compounds in petals and bacteria

The identification of volatile compounds released from petals and bacteria carrying *GmCCD4* and *gmicc1* was conducted following the methods previously described by Tian *et al*. (2014) and Zhang *et al*. (2015). The volatile compounds released in the headspace were analysed using headspace solid‐phase microextraction gas chromatography–mass spectrometry (SPME‐GC‐MS).

### Population genetics analysis and GWAS/Genetic diversity analysis

The original sequences of 182 cultivars were filtered using the Fastp software (Chen *et al*., 2018) and aligned with the reference genome of soybean (version: Gmax_275_Wm82.a2.v1) using the Burrows‐Wheeler Aligner (BWA) tool (Li and Durbin, 2009). SNP data (unpublished) were performed using the HapoltypeCaller method provided by GATK software (Mckenna *et al*., 2010) and used for the GWAS. SNPs with> 10% missing data or with minor allele frequencies (MAFs) of < 5% were removed. Genome‐wide association mapping was implemented in TASSEL following the mixed linear model (Bradbury *et al*., 2007). We used Storey and Tibshirani method (Storey and Tibshirani, 2003) to calculate Q value based on *p* value of GWAS analysis result. Then, the threshold value is determined by Q value ≤ 0.1. Finally, the threshold was set as *p *≤ 7.66×10^‐^^7^ (‐log_10_ *p* = 6.12).

## Conflict interest

The authors declare there is no conflict interest.

## Author contributions

X.Z.F. and S.X.Y. conceived and designed the research study, experiments and wrote the manuscript. J.S.G. conducted experiments and contributed to the experimental design under the supervision of X.Z.F. X.G. and B.L. participated in preparation of the *E. coli* strains engineered to accumulate β‐carotene. Lastly, K.Q.T., S.D.W. and G.L. analysed the genome data.

## Supporting information


**Figure S1** Mature seeds of the WT and the *gmicc1*, *2*, *3*, and *4* mutants, showing seed surfaces and seed cotyledons.
**Figure S2** Analysis of expression of*Glyma.01G154900.1* and genomic sequence in the WT and *gmicc* mutants.
**Figure S3** Detection of transgenic complementation plants.
**Figure S4** Expression of *GmCCD4* and synteny plot analysis.
**Figure S5** Phylogenetic analysis of the CCD and NCED proteins.
**Figure S6** Mass spectra and putative substrates.
**Figure S7** Frequency distributions of β‐carotenoid levels.
**Figure S8** β‐carotene content of the cultivars with different *GmCCD4*haplotypes, grouped based on the five non‐synonymous SNPs.
**Table S1** Average free carotenoid concentrations ± SD in the flowers and mature seeds of the WT, *gmicc1*, *2*, *3*, *4*mutants, and complementation lines (n = 5)
**Table S2** The F_1_ and F_2_ phenotype results of reciprocal crosses between mutants (*gmicc1*, *gmicc2*, *gmicc3*, and *gmicc4*), showing that these mutants are allelic.
**Table S3** Primers used in this study.
**Table S4** List of CCD genes.
**Table S5** β‐carotenoid content of soybean cultivars used in this study.
**Table S6** Comparison of the 36 SNPs in the genomic regions of *GmCCD4* from the 182 varieties of cultivated soybeans.
**Table S7** Amino acid hydrophobicity of the five SNPs.
