# Supplementary material for: GmCCD4 controls carotenoid content in soybeans
Source: Plant Biotechnol J. 2020 Nov 23;19(4):801–13. doi: 10.1111/pbi.13506 (PMC8051601; doi:10.1111/pbi.13506)
Supplement: Supplementary file 1 — Figure S1 Mature seeds of the WT and the gmicc1, 2, 3, and 4 mutants, showing seed surfaces and seed cotyledons. Figure S2 Analysis of expression ofGlyma.01G154900.1 and genomic sequence in the WT and gmicc mutants. Figure S3 Detection of transgenic complementation plants. Figure S4 Expression of GmCCD4 and synteny plot analysis. Figure S5 Phylogenetic analysis of the CCD and NCED proteins. Figure S6 Mass spectra and putative substrates. Figure S7 Frequency distributions of β‐carotenoid levels. Figure S8 β‐carotene content of the cultivars with different GmCCD4haplotypes, grouped based on the five non‐synonymous SNPs. Table S1 Average free carotenoid concentrations ± SD in the flowers and mature seeds of the WT, gmicc1, 2, 3, 4mutants, and complementation lines (n = 5) Table S2 The F1 and F2 phenotype results of reciprocal crosses between mutants (gmicc1, gmicc2, gmicc3, and gmicc4), showing that these mutants are allelic. Table S3 Primers used in this study. Table S4 List of CCD genes. Table S5 β‐carotenoid content of soybean cultivars used in this study. Table S6 Comparison of the 36 SNPs in the genomic regions of GmCCD4 from the 182 varieties of cultivated soybeans. Table S7 Amino acid hydrophobicity of the five SNPs. [file PBI-19-801-s001.docx]

**
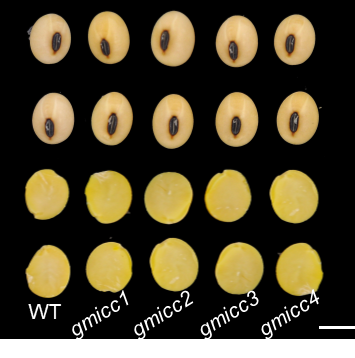
**

**Figure S1.** Mature seeds of the WT and the *gmicc1*, *2*, *3*, and *4* mutants, showing seed surfaces and seed cotyledons.

Scale bar = 5 mm.


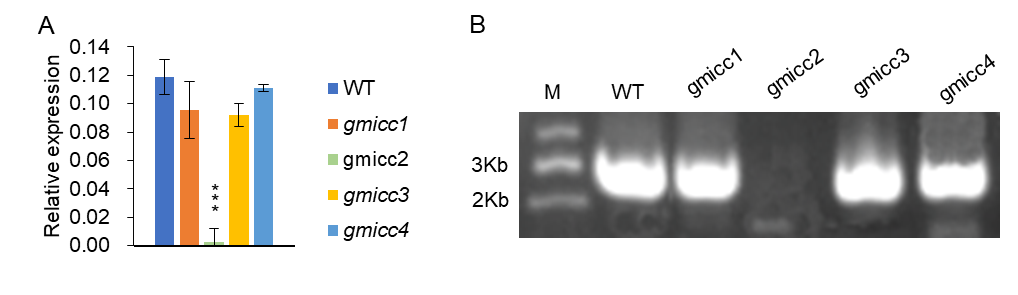


**Figure S2.** Analysis of expression of *Glyma.01G154900.1* and genomic sequence in the WT and *gmicc* mutants. **A.** Expression of *Glyma.01G154900.1* in unopened flowers of the WT and *gmicc* mutant plants. Bars represent mean ± SD of three biological replicates. **B.** PCR amplification of *Glyma.01G154900.1* from the WT, *gmicc1*, *gmicc2*, *3,* and *4* mutants, using primer OL6828. M, DNA maker. Asterisks indicate statistically signiﬁcant differences relative to the WT (***p < 0.001; Student's t test).


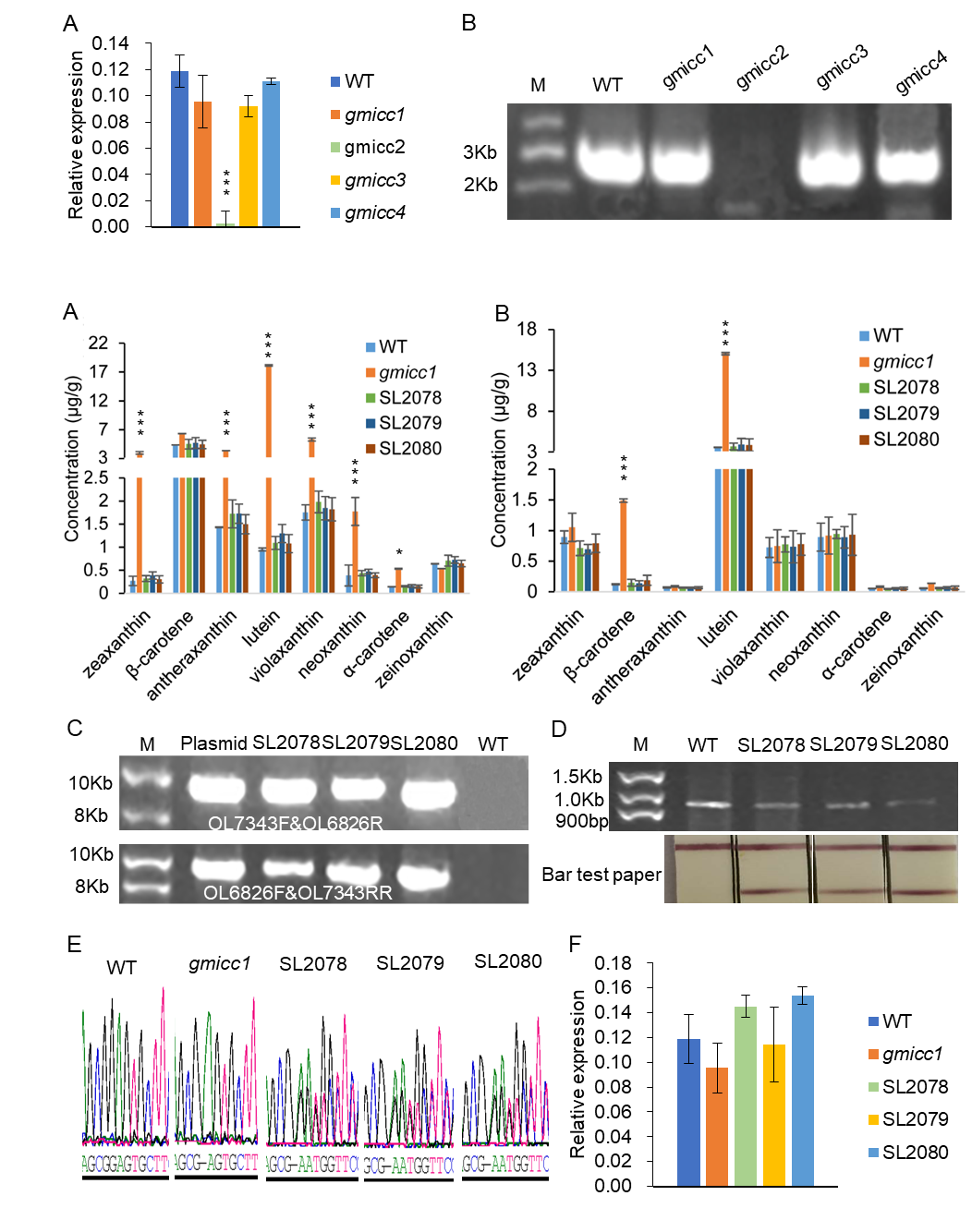


**Figure S3.** Detection of transgenic complementation plants. **A.** The carotenoid contents of the *gmicc1* and transgenic complementation flowers compared to the WT. Asterisks indicate statistically signiﬁcant differences relative to the WT (**p* < 0.05, ***p* < 0.01 and ****p* < 0.001; Student's *t* test). **B.** The carotenoid contents of the *gmicc1* and transgenic complementation seeds compared to the WT. Asterisks indicate statistically signiﬁcant differences relative to the WT (**p* < 0.05, ***p* < 0.01 and ****p* < 0.001; Student's *t* test). **C.** PCR amplification of the 7 kb *Glyma.01G154900.1* genomic fragment using primers of *Glyma.01G154900.1* and vector from plasmid of pCAMBIA3301: *Glyma.01G154900.1* (CK) and transgenic complementation plants. **D.** PAT/bar quick test of WT and transgenic complementation plants. PCR amplification of bar gene using primers OL7347 on the upper panel. **E.** Sequence analysis of *Glyma.01G154900.1* transcripts. A single nucleotide deletion exists in the *gmicc1* compared to WT, and two product peaks exist in the transgenic plants with wild and mutant sequences. **F.** Expression of *Glyma.01G154900.1* in unopened flowers of the WT and transgenic plants. Bars represent mean ± SD of three biological replicates.


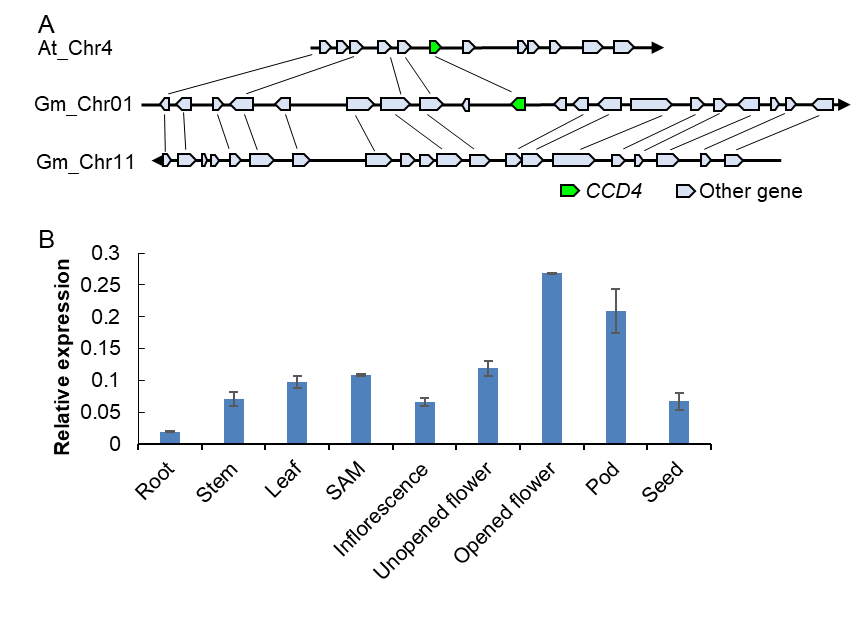


**Figure S4.** Expression of *GmCCD4* and synteny plot analysis. **A.** Synteny plot of soybean and *A. thaliana* sequence assemblies surrounding the *Arabidopsis CCD4* gene. Green arrows represent the anchor *CCD4* genes. Gray arrows indicate the genes flanking *CCD4*. Gene pairs conserved between segments are connected with lines. **B.** Expression of *GmCCD4* in roots, stems, leaves, SAMs, inflorescences, unopened flowers, opened flowers, pods, and seeds of the WT. Expression levels are presented as the mean ± SD of three biological replicates.


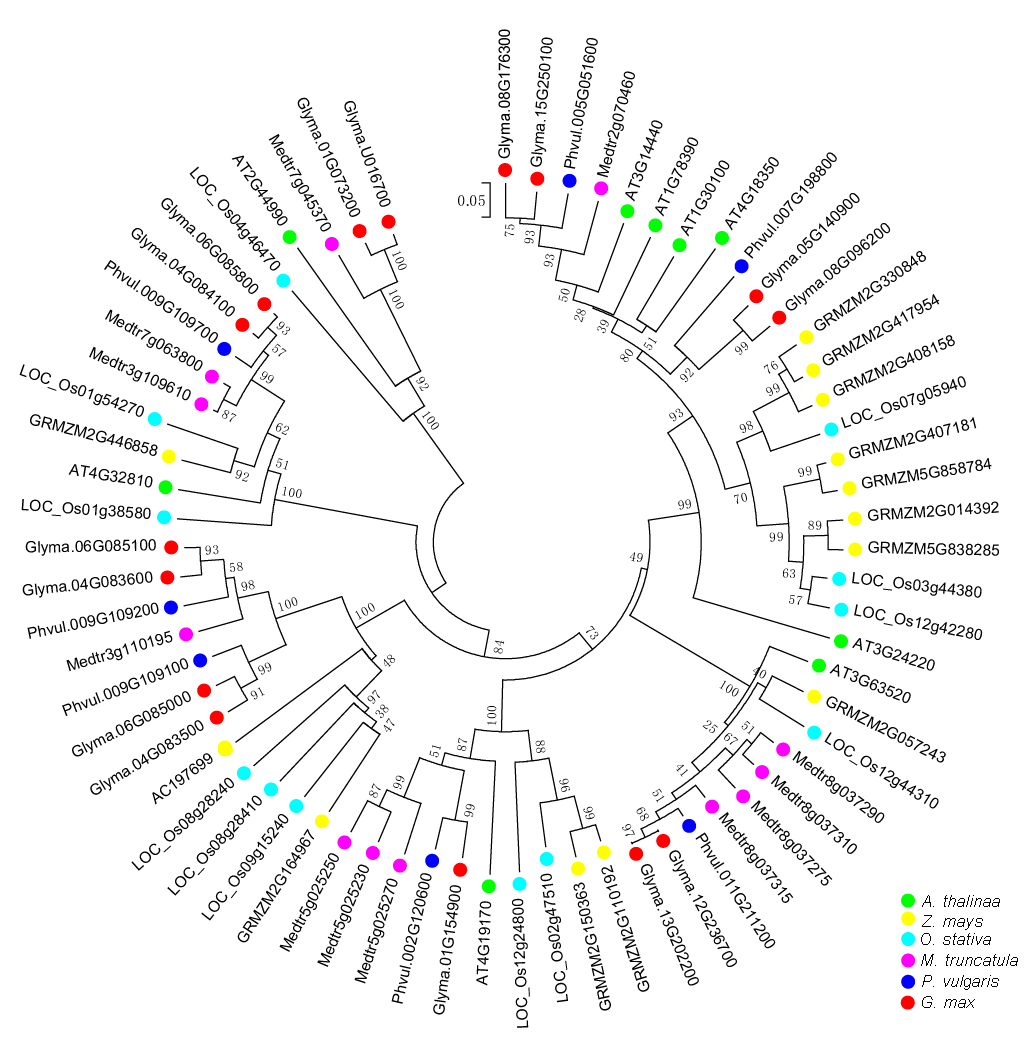


**Figure S5.** Phylogenetic analysis of the CCD and NCED proteins.


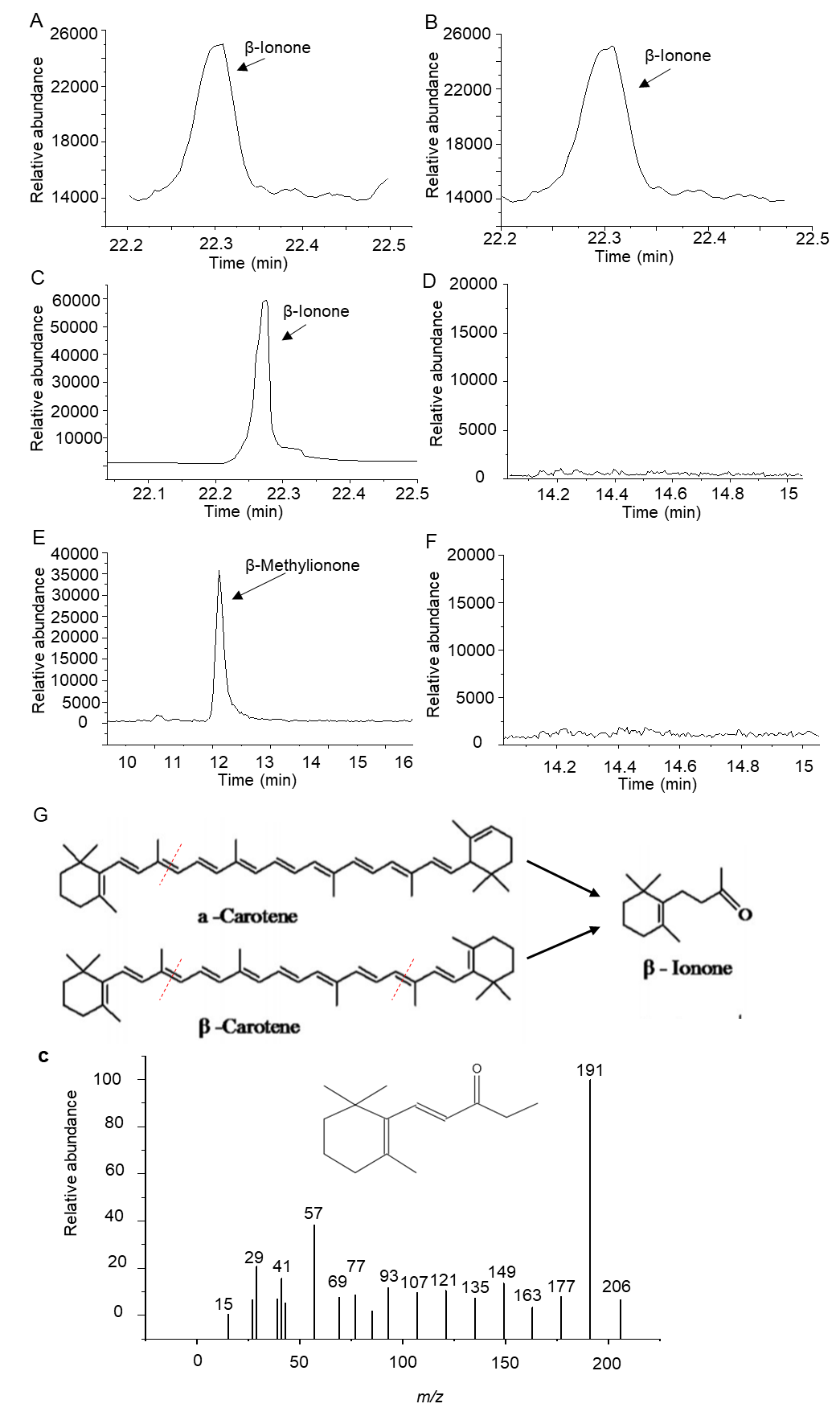


**Figure S6.** Mass spectra and putative substrates. **A-C.** Headspace-solid phase microextraction-GC-MS analysis of volatiles released from flower petals of transgenic complementation lines derived from the *gmicc1* mutant. **D-F.** GC-MS analysis of the headspaces of the cells bearing the pET32a plasmid (D), *GmCCD4* (E), or *gmicc1* (F). **G.** The carotenoid products catalyzed by GmCCD4.

**
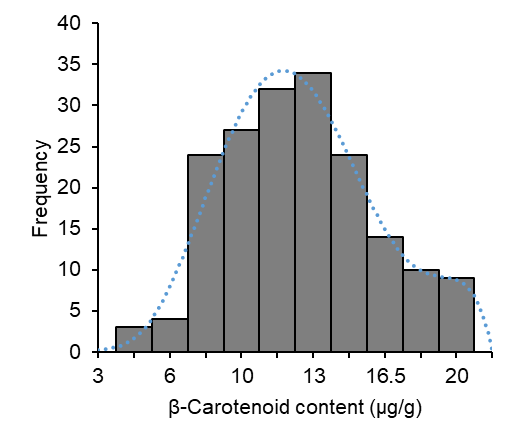
**

**Figure S7.** Frequency distributions of β-carotenoid levels.

**
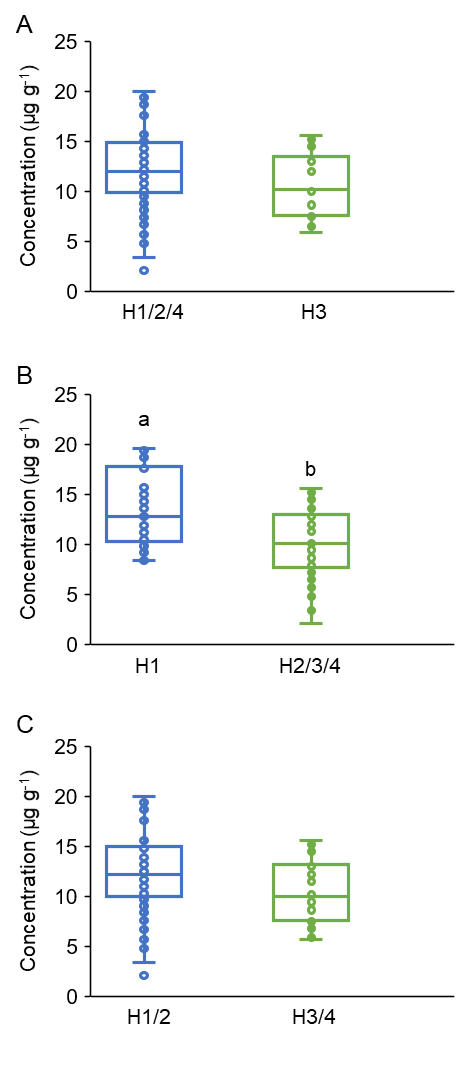
**

**Figure S8.** β-carotene content of the cultivars with different *GmCCD4* haplotypes, grouped based on the five non-synonymous SNPs.

Box edges indicate the interquartile range; whiskers indicate 1.5 × the interquartile range; and center lines indicate the median. Significant differences among groups were identified using one-way ANOVAs, followed by Tukey’s multiple comparisons post-hoc tests (*p* < 0.05). Different letters indicate distinct groups.

**Supplemental Tables:**

**Table S1.** Average free carotenoid concentrations ± SD in the flowers and mature seeds of the WT, *gmicc1, 2, 3, 4* mutants, and complementation lines (n = 5).

| Plant | Tissue | β-Carotene | Zeaxanthin | Antheraxanthin | Lutein | Violaxanthin | Neoxanthin | α-Catotene | Zeinoxanthin | Total |
| --- | --- | --- | --- | --- | --- | --- | --- | --- | --- | --- |
|  |  | (μg g^−1^) | (μg g^−1^) | (μg g^−1^) | (μg g^−1^) | (μg g^−1^) | (μg g^−1^) | (μg g^−1^) | (μg g^−1^) | (μg g^−1^) |
| Williams 82 | Seed | 0.123 ± 0.009 | 0.892 ± 0.103 | 0.071 ± 0.009 | 3.620 ± 0.032 | 0.724 ± 0.163 | 0.893 ± 0.227 | 0.054 ± 0.005 | 0.058 ± 0.007 | 6.435 ± 0.390 |
|  | Flower | 4.445 ± 0.498 | 0.269 ± 0.083 | 1.430 ± 0.267 | 0.950 ± 0.109 | 1.752 ± 0.164 | 0.388 ± 0.038 | 0.144 ± 0.013 | 0.640 ± 0.041 | 10.019 ± 0.198 |
| *gmicc1* | Seed | 1.490 ± 0.028 | 1.053 ± 0.231 | 0.090 ± 0.011 | 15.110 ± 0.141 | 0.746 ± 0.268 | 0.918 ± 0.301 | 0.084 ± 0.009 | 0.140 ± 0.002 | 19.620 ± 0.844 |
|  | Flower | 6.422 ± 0.478 | 2.986 ± 0.218 | 3.322 ± 0.358 | 19.069 ± 0.526 | 5.246 ± 0.419 | 1.773 ± 0.133 | 0.533 ± 0.060 | 0.537 ± 0.072 | 38.888 ± 1.357 |
| *gmicc2* | Seed | 1.740 ± 0.224 | 1.188 ± 0.153 | 0.088 ± 0.005 | 16.796 ± 1.027 | 0.806 ± 0.216 | 0.942 ± 0.081 | 0.090 ± 0.010 | 0.211 ± 0.037 | 20.674 ± 0.930 |
|  | Flower | 6.484 ± 0.506 | 3.701 ± 0.413 | 6.594 ± 0.491 | 19.578 ± 1.345 | 7.408 ± 0.428 | 3.042 ± 0.587 | 0.624 ± 0.068 | 0.816 ± 0.088 | 48.249 ± 1.444 |
| *gmicc3* | Seed | 1.276 ± 0.049 | 0.958 ± 0.007 | 0.075 ± 0.005 | 13.586 ± 0.995 | 1.053 ± 0.258 | 0.915 ± 0.222 | 0.066 ± 0.001 | 0.217 ± 0.041 | 17.505 ± 1.392 |
|  | Flower | 5.516 ± 0.416 | 1.977 ± 0.659 | 2.669 ± 0.358 | 16.118 ± 1.847 | 4.236 ± 0.654 | 1.978 ± 0.497 | 0.415 ± 0.111 | 0.460 ± 0.143 | 33.369 ± 1.719 |
| *gmicc4* | Seed | 1.473 ± 0.301 | 0.932 ± 0.087 | 0.074 ± 0.011 | 13.116 ± 0.990 | 0.746 ± 0.299 | 0.918 ± 0.337 | 0.084 ± 0.011 | 0.186 ± 0.040 | 16.268 ± 1.176 |
|  | Flower | 5.143 ± 1.069 | 1.710 ± 0.244 | 2.422 ± 0.557 | 15.393 ± 1.557 | 4.025 ± 0.616 | 1.910 ± 0.536 | 0.396 ± 0.034 | 0.453 ± 0.083 | 31.452 ± 1.026 |
| SL2078 | Seed | 0.148 ± 0.057 | 0.711 ± 0.120 | 0.068 ± 0.008 | 3.730 ± 0.412 | 0.773 ± 0.123 | 0.944 ± 0.073 | 0.048 ± 0.004 | 0.061 ± 0.012 | 6.484 ± 0.810 |
|  | Flower | 4.600 ± 0.831 | 0.320 ± 0063 | 1.722 ± 0.304 | 1.092 ± 0.140 | 1.982 ± 0.234 | 0.438 ± 0.058 | 0.152 ± 0.015 | 0.707 ± 0.118 | 11.013 ± 1.171 |
| SL2079 | Seed | 0.140 ± 0.042 | 0.700 ± 0.078 | 0.068 ± 0.008 | 5.136 ± 0.776 | 0.735 ± 0.263 | 0.890 ± 0.174 | 0.060 ± 0.010 | 0.057 ± 0.010 | 7.787 ± 1.369 |
|  | Flower | 4.804 ± 0.927 | 0.390 ± 0.075 | 1.726 ± 0.227 | 1.296 ± 0.406 | 1.845 ± 0.262 | 0.475 ± 0.110 | 0.158 ± 0.029 | 0.725 ± 0.141 | 11.420 ± 1.134 |
| SL2080 | Seed | 0.189 ± 0.082 | 0.793 ± 0.150 | 0.067 ± 0.016 | 3.901 ± 0.777 | 0.775 ± 0.178 | 0.930 ± 0.334 | 0.0594 ± 0.017 | 0.066 ± 0.027 | 6.781 ± 1.581 |
|  | Flower | 4.525±0.735 | 0.304±0.078 | 1.497±0.208 | 1.076±0.197 | 1.820±0.251 | 0.393±0.050 | 1.482±0.031 | 0.646±0.071 | 10.410±0.775 |

**Table S2.** The F_1_ and F_2_ phenotype results of reciprocal crosses between mutants (*gmicc1*, *gmicc2*, *gmicc3*, and *gmicc4*)*,* showing that these mutants are allelic.

|  | *gmicc1* (♂) | *gmicc2* (♂) | *gmicc3* (♂) | *gmicc4* (♂) |
| --- | --- | --- | --- | --- |
|  | F1 | | | |
| *gmicc1* (♀) | - | Hom (4 plants) | Hom (9 plants) | - |
| *gmicc2* (♀) | Hom (1 plants) | - | Hom (3 plants) | - |
| *gmicc3* (♀) | - | - | - | - |
| *gmicc4* (♀) | - | Hom (2 plants) | - | - |
|  | F2 | | | |
| *gmicc1* (♀) | - | Hom (40 plants) | Hom (40 plants) | - |
| *gmicc2* (♀) | Hom (40 plants) | - | Hom (40 plants) | - |
| *gmicc3* (♀) | - | - | - | - |
| *gmicc4* (♀) | - | Hom (40 plants) | - | - |

**Table S3.** Primers used in this study.

| Primers | Illustration | Locus | Forward sequence 5’→3’ |
| --- | --- | --- | --- |
|  |  |  | Reverse sequence 5’→3’ |
| MOL3492 | Marker | - | CGAGGATCCTCGCACCACGT |
|  |  |  | GTTCCAGCACTTCTTCTTCAGC |
| MOL3452 | Marker | - | CGAGTAGGAGACTGAGAACAAGTGG |
|  |  |  | ACTCCTCCCCTCAGCAAACTC |
| MOL1067 | Marker | - | CACGTAGCAGCCATCTTGGTTC |
|  |  |  | CTGTCTAAGGAATCCAGCCAAAAT |
| MOL1139 | Marker | - | TTGCACTAATAGCCTTAGTAGGAGTG |
|  |  |  | CATTATCCCCTTCCTTATATGCTGT |
| OL6826 | ProGmCCD4:GmCCD4 | *Glyma.01G154900.1* | CGAGCTCAAGTAGTGTTATAGTACAAAATTTGTACACC |
|  |  |  | CGGATCCAAGCAGACTCCGTGAACCTACAAAAATGT |
| OL6828 | Genomic *GmCCD4* | *Glyma.01G154900.1* | CCCAAGCTTGACAAGTGCAGCACACGATAAG |
|  |  |  | CGGGATCCCGACAACGACACTTTCCTCAG |
| OL7343 | pCMBIA3301 | *-* | GCAAGCTGCTCTAGCCAATAC  CTGGTCACCTGTAATTCACACG |
| OL7347 | Bar |  | ATGTAGTGGTTGACGATGGTGCAGA  GGTTTGCGTATTGGCTAGAG |
| OL15169 | RT-qPCR | *Glyma.01G154900.1* | CGTGATGCTAAGGATGAGTCGAT |
|  |  |  | TCTCCAGCGCGTGCTCCATCG |
|  |  |  | CCCAAGCTTTCACGACAACGACACTTT |
| OL6116 | *GmCCD4*/ | *Glyma.01G154900.1* | CGGAATTCATGGTCCCAAAACCAATC |
|  | *gmicc1* |  | CCCAAGCTTTCACGACAACGACACTTT |
| OL6117 | *CCD4* | *AT4G19170* | CGGAATTCATGGACTCTGTTTCTTCTTCTTC |
|  |  |  | ACGCGTCGACTTAAAGCTTATTAAGGTCACTTTC |
| OL6118 | *CCD1* | *AT3G63520* | CGGAATTCATGGCGGAGAAACTCAGTGATG |
|  |  |  | CGGGATCCTTATATAAGAGTTTGTTCCTGGAG |
| OL7151 | *GmCCD1-1* | *Glyma.13G202200.1* | TCACTGCCCCATCACTACCT |
|  |  |  | AGGCCCAACTCTGACAAACTC |
| OL7153 | *GmCCD1-2* | *Glyma.12G236700.1* | GATTGCTTGAATGGGGAGTTTGTC |
|  |  |  | TTGGTCCGACTCTGACAAACTC |
| OL7155 | *GmCCD7-1* | *Glyma.01G073200.* | CAGGCTACTCACCGTGTCTTG |
|  |  |  | G TATCCGTTACAGCCCAATCA |
| OL7157 | *GmCCD7-2* | *Glyma.U016700.1* | GGCATTCACGGATACCCACTAC |
|  |  |  | TGCCATACACTGCTGCCATT |
| OL7159 | *GmCCD8-1* | *Glyma.06G085800.1* | TCCAT AGTGATAGACCCTGAGACG |
|  |  |  | TGGGATGTGCGGAGTGAA |
| OL7161 | *GmCCD8-2* | *Glyma.04G084100.1* | GGCGATTATTGCCG ACTGT |
|  |  |  | CATCCAGTGGTATTCTGAACCG |
| OL7163 | *GmNCED2* | *Glyma.08G096200.1* | CCCAAAACCACTGACCCA |
|  |  | *Glyma.05G140900.1* | GTGCCAATAACGGGGAGAG |
| OL7167 | *GmNCED3-1* | *Glyma.15G250100.1* | GGAGGAACCCGAAAACGA |
|  |  |  | GCCTTATCTCAGACAAGACGCT |
| OL7165 | *GmNCED3-2* | *Glyma.08G176300.1* | TGCCATCGGTGAAACTCC |
|  |  |  | TCTTCGGACATGGCTAAGAGAT |
| OL10188 | *GmLUT5* | *Glyma.09G252800.1 Glyma.18G239900.1* | AGGCTTTACCCACAACCACC |
|  |  |  | CCATCTAATGCCCATCTTTCAG |
| OL10190 | *GmLUT1-1* | *Glyma.10G062900.1* | CTACGGCAAATACGCCAAGG |
|  |  |  | CAGCACATCTACAAAACACCCTATC |
| OL10192 | *GmLUT1-2* | *Glyma.13G147500.1* | GGTGAGTCTTGTAGCGTGGGA |
|  |  |  | GGAGAGGCGATAGGGTTTTGG |
| OL10194 | *GmCYP97B3* | *Glyma.11G016200.1* | TTTTGGGACCGCCCTGAT |
|  |  | *Glyma.01G226500.1* | AATCCGATATAACCTCGTTCGGAT |
| OL10196 | *GmZEP-1* | *Glyma.11G055700.1* | TGATAAGGCTTCATACCGTGTTAAG |
|  |  |  | CGGCTCCGACTTTTATGCTG |
| OL10198 | *GmZEP-2* | *Glyma.17G174500.1* | GCAAGATAATTCAGGCAGTTCAGT |
|  |  |  | CATGCTCACTCCGTAAATCAATC |
| OL10200 | *GmVDE* | *Glyma.03G253500.1 Glyma.19G251000.1* | TATACGCATCACGGCAGTAGC |
|  |  |  | TGGCCGCACAAGATGGGTT |
| OL10202 | *GmNSY* | *Glyma.12G186300.1* | CTATTGGTAGCACTTCTGGGGTAG |
|  |  |  | CCCTGACTAGCCTGTTCTCAATT |
| OL6553 | *GmCons4* | *Glyma.12G020500* | GATCAGCAATTATGCACAACG |
|  |  |  | CCGCCACCATTCAGATTATGT |

**Table S4.** List of CCD genes.

| Number | Species | Gene symbol | Locus tag |
| --- | --- | --- | --- |
| 1 | *Arabidopsis* | *CCD7* | AT2G44990 |
| 2 |  | *CCD1* | AT3G63520 |
| 3 |  | *CCD4* | AT4G19170 |
| 4 |  | *CCD8* | AT4G32810 |
| 5 | *Glycine max* | *CCD7* | Glyma.01G073200 |
| 6 |  | *CCD4* | Glyma.01G154900 |
| 7 |  | *CCD8* | Glyma.04G084100 |
| 8 |  | *CCD8* | Glyma.06G085800 |
| 9 |  | *CCD1* | Glyma.12G236700 |
| 10 |  | *CCD1* | Glyma.13G202200 |
| 11 |  | *CCD7* | Glyma.U016700 |
| 12 | *Zea mays* | *CCD1* | GRMZM2G057243 |
| 13 |  | *CCD4* | GRMZM2G110192 |
| 14 |  | *CCD4* | GRMZM2G150363 |
| 15 |  | *CCD8* | GRMZM2G446858 |
| 16 | *Oryza sativa* | *CCD8* | LOC_Os01g38580 |
| 17 |  | *CCD8* | LOC_Os01g542700 |
| 18 |  | *CCD4* | LOC_Os02g47510 |
| 19 |  | *CCD7* | LOC_Os04g46470 |
| 20 |  | *CCD4* | LOC_Os12g24800 |
| 21 |  | *CCD1* | LOC_Os12g44310 |
| 22 | *Medicago truncatula* | *CCD8* | Medtr3g109610 |
| 23 |  | *CCD4* | Medtr5g025230 |
| 24 |  | *CCD4* | Medtr5g025250 |
| 25 |  | *CCD4* | Medtr5g025270 |
| 26 |  | *CCD7* | Medtr7g045370 |
| 27 |  | *CCD8* | Medtr7g063800 |
| 28 |  | *CCD1* | Medtr8g037275 |
| 29 |  | *CCD1* | Medtr8g037290 |
| 30 |  | *CCD1* | Medtr8g037310 |
| 31 |  | *CCD1* | Medtr8g037315 |
| 32 | *Phaseolus vulgaris* | *CCD4* | Phvul.002G120600 |
| 33 |  | *CCD8* | Phvul.009G109700 |
| 34 |  | *CCD1* | Phvul.011G211200 |
| 35 | *Vitis vinifera* | *CCD7* | GSVIVG01018217001 |
| 36 |  | *CCD4* | GSVIVG01024318001 |
| 37 |  | *CCD1* | GSVIVG01032103001 |
| 38 |  | *CCD1* | GSVIVG01032110001 |
| 39 |  | *CCD8* | GSVIVG01035626001 |
| 40 |  | *CCD4* | GSVIVG01036862001 |
| 41 | *Lotus japonicus* | *CCD7* | ADM88552 |
| 42 | *Physcomitrella patens* |  | Pp3c25_4816V3.1 |

The genes in this table are classified according to Figure 3A.

**Table S5.** β-carotenoid content of soybean cultivars used in this study.

| Haplotype | Name | β-carotenoid concentration (μg g^-1^) |
| --- | --- | --- |
| H1 | Beijiang 1 | 7.57±0.04 |
|  | Dongnong 63 | 7.57±0.19 |
|  | Henong 61 | 7.63±0.08 |
|  | Hefeng 48 | 7.69±0.04 |
|  | Dongnong 48 | 7.82±0.08 |
|  | Dengke 1 | 7.88±0.26 |
|  | Dongnong 52 | 7.88±0.16 |
|  | Hefeng 55 | 8.00±0.08 |
|  | Heinong 54 | 8.03±0.12 |
|  | Heinong 50 | 8.22±0.12 |
|  | Henong 62 | 8.40±0.04 |
|  | Kenjiandou 27 | 8.40±0.08 |
|  | Henong 92 | 8.49±0.08 |
|  | Huajiang 2 | 8.64±0.07 |
|  | Heihe 53 | 8.73±0.07 |
|  | Beidou 10 | 8.80±0.18 |
|  | Heihe 45 | 8.86±0.15 |
|  | Mengdou 36 | 9.17±0.04 |
|  | Heinong 55 | 9.26±0.11 |
|  | Heihe 35 | 9.41±0.04 |
|  | Suinong 37 | 9.44±0.08 |
|  | Kenfeng 20 | 9.63±0.04 |
|  | Rundou 1 | 9.66±0.07 |
|  | Suinong 36 | 9.69±0.26 |
|  | Suinong 35 | 9.75±0.07 |
|  | Beidou 53 | 9.81±0.34 |
|  | Heihe 44 | 10.0±0.04 |
|  | Shengdou 44 | 10.0±0.08 |
|  | Beidou 42 | 10.2±0.15 |
|  | Heihe 43 | 10.2±0.11 |
|  | Suinong 22 | 10.2±0.11 |
|  | Jiashi A | 10.3±0.07 |
|  | Kennong 32 | 10.3±0.13 |
|  | Dongnong 55 | 10.3±0.04 |
|  | Huajiang 4 | 10.3±0.04 |
|  | Kendou 25 | 10.3±0.11 |
|  | Henong 76 | 10.4±0.07 |
|  | Dongnong 56 | 10.5±0.04 |
|  | Hefeng 50 | 10.5±0.15 |
|  | Hefeng 35 | 10.8±0.04 |
|  | Beidou 14 | 11.0±0.07 |
|  | Beidou 37 | 11.0±0.13 |
|  | Hefeng 25 | 11.1±0.19 |
|  | Dongnong 7 | 11.1±0.11 |
|  | Nengao 3 | 11.2±0.07 |
|  | Jidadou 1 | 11.2±0.04 |
|  | Dongnong 44 | 11.2±0.11 |
|  | Huajiang 17 | 11.3±0.13 |
|  | Jiamidou 6 | 11.5±0.04 |
|  | Suinong 27 | 11.5±0.08 |
|  | Jinyuan 55 | 11.6±0.07 |
|  | Henong 75 | 11.7±0.04 |
|  | Jinshan 1 | 11.7±0.04 |
|  | Beidou 5 | 11.8±0.26 |
|  | Henong 97 | 11.9±0.19 |
|  | Beidou 40 | 12.1±0.24 |
|  | Kenfeng 17 | 12.2±0.15 |
|  | Beidou 33 | 12.2±0.07 |
|  | Beidou 51 | 12.5±0.11 |
|  | Mengdou 33 | 12.5±0.08 |
|  | Beidou 31 | 12.8±0.07 |
|  | Hefeng 39 | 12.8±0.75 |
|  | Suinog 10 | 12.9±0.27 |
|  | Suinong 48 | 13.0±0.04 |
|  | Beidou 29 | 13.2±0.15 |
|  | Dongnong 42 | 13.2±0.08 |
|  | Heihe 48 | 13.2±0.17 |
|  | Huajiang 5 | 13.2±0.04 |
|  | Dongnong 42A | 13.3±0.23 |
|  | Beidou 36 | 13.4±0.18 |
|  | Hefeng 51 | 13.6±0.11 |
|  | Haojiang 1 | 13.9±0.11 |
|  | Suinong 26 | 14.1±0.07 |
|  | Jinong 28 | 14.2±0 |
|  | Heinong 35 | 14.3±0.15 |
|  | Jiyu 64 | 14.4±0.04 |
|  | Shangning 14 | 14.8±0.04 |
|  | Hefeng 57 | 14.9±0.07 |
|  | Suinong 39 | 14.9±0.04 |
|  | Kennong 33 | 15.0±0.15 |
|  | Suinong 49 | 15.1±0.11 |
|  | Zhongzuo 13030 | 15.4±0.11 |
|  | Suinong 53 | 15.6±0.11 |
|  | Jinong 41 | 15.7±0.39 |
|  | Suinong 44 | 17.6±0.13 |
|  | Beidou 43 | 17.6±0.07 |
|  | Suinong 14 | 17.8±0.08 |
|  | Dongsheng 25 | 17.8±0.11 |
|  | Jiunong 26 | 17.8±0.08 |
|  | Suinong 76 | 17.8±0.30 |
|  | Dongsheng 3 | 17.9±0.04 |
|  | Dongsheng 9 | 17.9±0.08 |
|  | Songsheng 6 | 17.9±0.08 |
|  | Xuzhou 0705 | 17.9±0.27 |
|  | Suinong 41 | 17.9±0.15 |
|  | Suinong 75 | 18.0±0.04 |
|  | Dongsheng 20 | 18.0±0.18 |
|  | Dongsheng 5 | 18.0±0.11 |
|  | Dongsheng 12 | 18.1±0.18 |
|  | Jiyu 93 | 18.1±0.04 |
|  | Dongsheng 10 | 18.1±0.04 |
|  | Dongsheng 16 | 18.1±0.08 |
|  | Jida 131 | 18.1±0.11 |
|  | Dongsheng 17 | 18.2±0.08 |
|  | Dongsheng 18 | 18.7±0.04 |
|  | Wandou 38 | 18.8±0.11 |
|  | Shanbeiheidou | 18.8±0.04 |
|  | Ludou 10 | 18.8±0.15 |
|  | Fendou 93 | 18.9±0.04 |
|  | Taicheng 126 | 19.1±0.04 |
|  | Jin 22 | 19.4±2.24 |
|  | Jiadou 25 | 19.6±0.18 |
|  | Kenjian 35 | 20.0±0.08 |
| H2 | Wandou 35 | 2.06±0.11 |
|  | Dongdou 82 | 3.35±0.04 |
|  | Heinong 46 | 4.77±0.13 |
|  | Hefeng 52 | 5.63±0.24 |
|  | Jilin 32 | 6.67±0.04 |
|  | Jiyu 202 | 7.13±0.04 |
|  | Hudou 15 | 7.29±0.04 |
|  | Hefeng 40 | 7.60±0.04 |
|  | Kenfeng 16 | 7.69±0.04 |
|  | Dongnong 47 | 8.33±0.28 |
|  | Heinong 69 | 9.17±0.04 |
|  | Heinong 66 | 9.75±0.07 |
|  | Hefeng 42 | 9.87±0.04 |
|  | Kendou 40 | 9.97±0.04 |
|  | Heinong 67 | 10.1±0.08 |
|  | Jiyu 86 | 12.0±0.04 |
|  | Henong 70 | 12.2±0.04 |
|  | Jihuang 28 | 7.90±0.39 |
|  | Henong 48 | 13.0±0.04 |
|  | Kendou 39 | 13.2±0.07 |
|  | Jihuang 12 | 9.01±0.15 |
|  | Dongsheng 103 | 9.13±0.33 |
|  | Dongsheng 105 | 11.4±0.32 |
|  | Huachengdou 8 | 11.3±0.08 |
|  | Shangning 17 | 11.3±0.04 |
|  | Dongsheng 101 | 12.0±0.11 |
|  | Qindou 2018 | 12.3±0.15 |
|  | Dongsheng 22 | 13.1±0.15 |
|  | Meng 5803 | 13.2±0.30 |
|  | Dong 064 | 13.2±0.37 |
|  | Changnong 20 | 13.3±0.15 |
|  | Jiyu 508 | 12.8±0.27 |
|  | Jiyu 72 | 13.4±0.04 |
|  | Jidadou 2 | 12.9±0.04 |
|  | Henong 41 | 12.8±0.04 |
|  | Jiyu 406 | 12.6±0.00 |
| H3 | Dongnong 60 | 5.84±0.07 |
|  | Andou 1478 | 6.42±0.15 |
|  | Kennog 30 | 6.79±0.08 |
|  | Haojiang 13 | 6.94±0.04 |
|  | Henong 59 | 7.03±0.04 |
|  | Hefeng 60 | 7.49±0.04 |
|  | Keshan 1 | 7.54±0.08 |
|  | Hefeng 45 | 7.66±0.13 |
|  | Pingandou 8 | 8.60±0.04 |
|  | Suinong 30 | 9.24±0.04 |
|  | Henong 67 | 9.99±0.04 |
|  | Hefeng 7 | 10.0±0.08 |
|  | Jiling 39 | 10.2±0.08 |
|  | Jidadou 3 | 11.9±0.27 |
|  | Henong 63 | 13.0±0.04 |
|  | Dong 06A | 15.0±0.04 |
|  | Henong 71 | 15.5±0.08 |
|  | Jihuang 105 | 15.5±1.30 |
|  | Xinjiangdadou | 14.5±0.04 |
|  | Hefeng 58 | 16.6±4.26 |
|  | Dong 06A3 | 13.6±0.07 |
|  | Jidadou 5 | 13.1±0.11 |
|  | Xuzhou 0701 | 13.4±0.33 |
|  | Zheng 95112 | 13.1±0.07 |
|  | Jinong 18 | 13.3±0.27 |
| H4 | Dian cha Dou | 5.63±0.04 |
|  | Changjihuangdou | 7.35±0.07 |
|  | Hudou9765 | 8.12±0.04 |
|  | Tachenghuangdou 021 | 8.98±0.15 |
|  | Dongyu | 9.41±0.08 |
|  | Dong 41 | 11.5±0.07 |
|  | Nong 28 | 12.2±0.04 |
|  | Tacheng 124 | 12.5±0.07 |

**Table S6.** Comparison of the 36 SNPs in the genomic regions of *GmCCD4* from the 182 varieties of cultivated soybeans.

| POS | 49242529 | 49242913 | 49242932 | 49242946 | 49242948 | 49242954 | 49242976 |  | 49242978 |  | 49243009 | 49243071 | 49243102 | 49243147 | 49243155 | 49243168 | 49243176 | 49243178 | 49243214 | 49243226 | 49243252 | 49243257 | 49243281 | 49243287 | 49243307 | 49243333 | 49243342 | 49243481 | 49243695 | 49243719 | 49243765 | 49243815 | 49243874 | 49243881 | 49244383 | 49244461 | 49244470 | 49244606 |
| --- | --- | --- | --- | --- | --- | --- | --- | --- | --- | --- | --- | --- | --- | --- | --- | --- | --- | --- | --- | --- | --- | --- | --- | --- | --- | --- | --- | --- | --- | --- | --- | --- | --- | --- | --- | --- | --- | --- |
|  | Exon2 | Intron1 | Intron1 | Intron1 | Intron1 | Intron1 | Intron1 | Intron1 | Intron1 |  | Intron1 | Intron1 | Intron1 | Intron1 | Intron1 | Intron1 | Intron1 | Intron1 | Intron1 | Intron1 | Intron1 | Intron1 | Intron1 | Intron1 | Intron1 | Intron1 | Intron1 | Exon1 | Exon1 | Exon1 | Exon1 | Exon1 | Exon1 | Exon1 | Exon1 | Exon1 | Exon1 | 5’UTR |
| REF | T | C | T | G | C | T | C |  | G |  | G | T | A | A | C | A | C | A | G | T | C | C | G | A | T | C | T | A | G | G | A | G | A | A | A | G | A | G |
| ALT | C | A | G | T | A | C | G |  | T |  | T | C | T | G | A | C | T | G | T | C | T | T | A | G | G | G | C | T | A | A | G | A | C | C | T | A | G | A |
| H1 | TT | AA | GG | TT | AA | CC | GG |  | TT |  | TT | CC | TT | GG | AA | CC | TT | GG | TT | CC | TT | TT | AA | GG | GG | GG | CC | TT | GG | AA | GG | GG | CC | CC | TT | AA | GG | AA |
| H2 | CC | CC | TT | GG | CC | TT | CC |  | GG |  | GG | TT | AA | AA | CC | AA | CC | AA | GG | TT | CC | CC | GG | GG | TT | CC | TT | TT | AA | GG | GG | AA | CC | CC | AA | GG | GG | GG |
| H3 | TT | CC | TT | GG | CC | TT | CC |  | GG |  | GG | TT | AA | AA | CC | AA | CC | AA | GG | TT | CC | CC | GG | AA | TT | CC | TT | AA | GG | GG | AA | GG | AA | AA | AA | GG | AA | GG |
| H4 | CC | CC | TT | GG | CC | TT | CC |  | GG |  | GG | TT | AA | AA | CC | AA | CC | AA | GG | TT | CC | CC | GG | AA | TT | CC | TT | AA | GG | GG | AA | GG | AA | AA | AA | GG | GG | GG |

**Table S7.** Amino acid hydrophobicity of the five SNPs.

|  | SNP1 | SNP2 | SNP3 | SNP4 | SNP5 |
| --- | --- | --- | --- | --- | --- |
| Location | 49244470 | 49244461 | 49244383 | 49243874 | 49243481 |
| Ref | Leu (3.8) | Ala (1.8) | Ile (4.5) | Phe (2.8) | Leu (3.8) |
| Alt | Pro (−1.6) | Val (4.2) | Lys (−3.9) | Val (4.2) | Met (1.9) |

Numbers in brackets are amino acid hydrophobic parameters.
